# The evolution of giant flightless birds and novel phylogenetic relationships for extinct fowl (Aves, Galloanseres)

**DOI:** 10.1098/rsos.170975

**Published:** 2017-10-11

**Authors:** Trevor H. Worthy, Federico J. Degrange, Warren D. Handley, Michael S. Y. Lee

**Affiliations:** 1 College of Science and Engineering, Flinders University, Adelaide, South Australia, Australia; 2 Centro de Investigaciones en Ciencias de la Tierra (CICTERRA), UNC, CONICET, Av. Vélez Sársfield 1611, X5016GCA, Córdoba, Argentina; 3 Earth Sciences Section, South Australian Museum, North Terrace, Adelaide, South Australia, Australia

**Keywords:** Galloanseres, Dromornithidae, *Gastornis*, *Brontornis*, fossil birds, morphological phylogenetics

## Abstract

The extinct dromornithids, gastornithids and phorusrhacids are among the most spectacular birds to have ever lived, with some giants exceeding 500 kg. The affinities and evolution of these and other related extinct birds remain contentious, with previous phylogenetic analyses being affected by widespread convergence and limited taxon sampling. We address these problems using both parsimony and tip-dated Bayesian approaches on an expansive taxon set that includes all key extinct flightless and flighted (e.g. *Vegavis* and lithornithids) forms, an extensive array of extant fowl (Galloanseres), representative Neoaves and palaeognaths. The Paleogene volant Lithornithidae are recovered as stem palaeognaths in the Bayesian analyses. The Galloanseres comprise four clades inferred to have diverged in the Late Cretaceous on Gondwana. In addition to Anseriformes and Galliformes, we recognize a robust new clade (Gastornithiformes) for the giant flightless Dromornithidae (Australia) and Gastornithidae (Eurasia, North America). This clade exhibits parallels to ratite palaeognaths in that flight presumably was lost and giant size attained multiple times. A fourth clade is represented by the Cretaceous *Vegavis* (Antarctica), which was strongly excluded from Anseriformes; thus, a crucial molecular calibration point needs to be reconsidered. The presbyornithids *Wilaru* (Australia) and *Presbyornis* (Northern Hemisphere) are robustly found to be the sister group to Anatoidea (Anseranatidae + Anatidae), a relatively more basal position than hitherto recognized. South America's largest bird, *Brontornis*, is not a galloansere, but a member of Neoaves related to Cariamiformes; therefore, giant Galloanseres remain unknown from this continent. Trait analyses showed that while gigantism and flightlessness evolved repeatedly in groups, diet is constrained by phylogeny: all giant Galloanseres and palaeognaths are herbivores or mainly herbivorous, and giant neoavians are zoophagous or omnivorous.

## 1. Introduction

Landfowl (Galliformes) and waterfowl (Anseriformes) form a diverse and important clade (Galloanseres) that is sister to Neoaves (all other extant non-palaeognath birds). Taken together, all constitute the Neognathae which is sister to remaining extant birds, the Palaeognathae (e.g. [1–3]). Extant Galloanseres include medium to large, usually volant birds grouped in three waterfowl and five landfowl families. However, three extinct families greatly expand the group's morphological diversity to include giant terrestrial, flightless forms which include some of the largest birds ever. These are the mihirungs (Dromornithidae) from Australia with eight described species [4–7] and the Gastornithidae of Eurasia and North America, both long considered to have anseriform affinities [8–16], and the Melanesian Sylviornithidae, with likely galliform affinities [17]. Another group of giant flightless birds with potential affinities to Galloanseres are the Brontornithidae from South America. The single species in this family, *Brontornis burmeisteri* Moreno and Mercerat, 1891 [18], has usually been considered as a phorusrhacid neoavian, albeit an unusual one (e.g. [19]), but following a proposal that it has galloansere affinities near Anseriformes [20], Brontornithidae has been placed within Galloanseres [21–24].

The phylogenetic relationships of all these giant flightless Galloanseres—to each other and to living birds—have not been robustly resolved. Andors [12] analysed *Gastornis* (then *Diatryma*) in the context of Galliformes, Anhimidae, Anseranatidae and Anatidae, finding it was the sister group of the anseriforms. Murray & Vickers-Rich [6] used Andors' matrix and marshalled various other evidence to show that dromornithids were anseriforms, but were unable to further constrain their relationships with regard to the extant families Anhimidae, Anseranatidae and Anatidae. Agnolin [20] found that Dromornithidae, *Brontornis* and *Gastornis* (as *Diatryma*) formed successive sister groups to Anseriformes in a limited analysis of 51 characters with higher-level terminal taxa (genera or families). Mayr [25], while assessing the family-group relationships of the aberrant group Pelagornithidae (bony-toothed birds) in the context of a range of families, some coded as family-level taxa from the literature, found a more basal relationship for Dromornithidae and Sylviornithidae: as successive sister-groups to all extant Galloanseres. Using Mayr & Clarke's [26] matrix, Degrange *et al*. [23] found that *Brontornis* was an anseriform related to the screamers (Anhimidae). Most recently, Worthy *et al*. [17] found support for the galloansere affinity of *Dromornis planei* Rich, 1979 [4], *Sylviornis neocaledoniae* Poplin, 1980 [27] and *Megavitiornis altirostris* Worthy, 2000 [28], which all emerged as stem galliforms. Only in the latter analysis was a representative sample of extant Galloanseres included, along with adequate outgroup taxa (Neoaves and palaeognaths) and other significant fossils. Nevertheless, this uncertainty in how these extinct groups of Galloanseres were related to each other and to extant taxa remains a significant problem and as yet no analyses have included all relevant taxa in a single comprehensive phylogenetic analysis.

These giant flightless Galloanseres show striking morphological convergence with flightless palaeognaths (ratites), especially the large extinct Aepyornithidae (elephant birds; Madagascar) and Dinornithiformes (moa; New Zealand). All share massive hind limbs and greatly reduced pectoral girdle elements (wing bones), fusion of coracoid and scapula, and have a sternum lacking a carina. The Australian dromornithids were long considered to be palaeognaths [4,29] until studies of skull material precluded palaeognath affinities [5,30,31]. The combination of enlarged hind limbs and markedly reduced pectoral elements has plagued resolution of the phylogenetic relationships of the ratite palaeognaths using morphological data (e.g. [32–35]). Only with rich molecular data have the relationships of the extant and the recently extinct palaeognaths been resolved. These relationships bear little similarity to any derived from morphological data, such as the sister-group relationships of moa and tinamou, or that of kiwi and elephant birds [36–39]. Therefore, it is likely that untangling the evolutionary history of the giant flightless Galloanseres will present similar challenges. Nevertheless, attempting to understand their relationships to extant Galloanseres is important for its potential to shed light on when and how Galloanseres diverged from Neoaves. Moreover, establishing what characterizes the ancestral state of Neognathae may facilitate the recognition of stem-neognath fossils, which have so far eluded detection, but are vital to understanding the evolution of crown-group Aves.

While the initial aim of this study was to determine the position of the Australian Dromornithidae within Galloanseres and their relationship to other giant Galloanseres, this also required robustly resolving the relationships of several geologically ancient volant taxa. These include the Late Cretaceous (69–66 Ma) *Vegavis iaai* Clarke *et al.*, 2005 [40], the Eocene presbyornithids and the Paleogene lithornithid palaeognaths. All have the potential to inform on the morphology of the basal ancestral nodes within Aves, and so influence phylogeny and character optimization. *Vegavis iaai* is presently considered a member of crown group Anseriformes [17,40–42], but its relationships therein are not strongly resolved. Likewise, the enigmatic presbyornithids are considered to be the sister group to Anatidae within Anseriformes [43–45]. However, when Anseriformes were analysed alongside representative Galliformes and other galloansere taxa, including *Vegavis*, *Presbyornis* formed a clade with *Anseranas*, in a trichotomy with Anatidae and *Vegavis* [17, fig. 13].

The young (less than 80 Ma) age of crown-group palaeognaths and their interrelationships—with New Zealand moa sister to (volant) South American tinamous and New Zealand kiwi sister to Madagascan elephant birds—indicates that flightless ratites had volant ancestors, attaining modern distributions by recent dispersal rather than by ancient vicariance [36,37,39]. In this context, the volant Paleocene/Eocene Northern Hemisphere lithornithids are important. They are considered to be palaeognaths [46], but their relationships to the crown group are controversial. The two most recent phylogenetic analyses to examine them obtained very divergent results. Nesbitt & Clarke [35] recovered lithornithids as the sister group of tinamous in unconstrained analyses; however, in various analyses where extant palaeognath relationships were constrained to topologies reflecting molecular results, lithornithids were found to be sister to all extant palaeognaths. Problematically, Yonezawa *et al.* [39] stated that this result was compromised by the fact that Nesbitt & Clarke's [35] constraints ‘implicitly assumed the monophyly of extant palaeognaths’ and that without this constraint, lithornithids group with tinamous. In analyses of another dataset, Worthy *et al*. [17] found that lithornithids remained as the sister group of tinamous, even when extant palaeognaths were constrained to the molecular topology. Resolution of lithornithid relationships is, therefore, as for *Vegavis*, critical in establishing the morphological characteristics of the basal nodes within Aves such as Neornithes, Neognathae and Galloanseres.

The aim of this contribution is therefore to resolve the phylogenetic relationships and evolution of the giant flightless Galloanseres in a comprehensive taxon set that includes representative galliforms, anseriforms, Neoaves and palaeognaths, and relevant flightless and volant fossil taxa. This will also facilitate recognition of the characteristics of stem neognaths which may allow the identification of their fossils.

## 2. Material and methods

### 2.1. Nomenclature

For extant birds, we follow the nomenclature in Dickinson & Remsen [47]. Names for specific bone landmarks follow Baumel & Witmer [48] unless otherwise indicated.

### 2.2. Material

The material used in the scoring of characters is listed in Worthy *et al*. [17]. Additional fossil taxa and specimens used are listed in the electronic supplementary material.

### 2.3. Morphological, body size, ecological and stratigraphic data

The data matrix used by Worthy *et al*. [17] was modified by adding several new characters and substantially extending taxon sampling. Five new characters (286–290) were added, and several other characters were slightly modified with the addition of new states and/or correction of some codings; 60 characters forming clear morphoclines were ordered (see the electronic supplementary material).

Worthy *et al*.'s [17] matrix had 37 taxa including seven palaeognath outgroup taxa. Here, we have modified this taxon set by the addition of a fourth extant species in Neoaves, *Cariama cristata*, the presumed living relative of *Patagornis* and possibly *Brontornis*. We excluded *Mwalau* as this megapodiid has considerable missing data and its inclusion reduced resolution in Galliformes, and was not central to the question of how dromornithids relate to the major clades of Galloanseres. We retained the sylviornithids *Sylviornis* and *Megavitiornis* as they were found to be the sister group to crown Galliformes [17] and therefore one of the basally diverging Galloanseres. Moreover, they share with dromornithids intriguing similarities in cranial morphology, particularly of the bill.

Several additional fossil taxa were added to the matrix to address the precise relationships of dromornithids and other key Galloanseres mentioned above. The following dromornithid species were added to complement *D. planei* and capture the diversity of this group: *Genyornis newtoni* Stirling and Zeitz, 1896 [49], *D. stirtoni* Rich, 1979 [4], *D. murrayi* Worthy *et al.*, 2016 [7], *Ilbandornis woodburnei* Rich, 1979 [4], *I. lawsoni* Rich, 1979 [4], *Barawertornis tedfordi* Rich, 1979 [4], see Worthy *et al.* [7]. The gastornithids *Gastornis giganteus* (Cope, 1876) [50] and *G. parisiensis* Hébert, 1855 [51] were chosen because of relative completeness to represent Gastornithidae [8,10,13–16]. We added the newly recognized austral presbyornithid *Wilaru tedfordi* Boles *et al.*, 2013 [52], so its affinities could be assessed within this comprehensive dataset, which also includes *Burhinus* in whose family *W. tedfordi* was originally described. Moreover, in the redescription of *W. tedfordi*, De Pietri *et al.* [53] noted that it shared more similarities with the Argentine *Telmabates antiquus* Howard, 1955 [54] than with *Presbyornis pervetus* Wetmore, 1926 [55], thereby raising the possibility of Northern and Southern Hemisphere presbyornithid radiations. To represent Phorusrhacidae, the middle-sized Miocene *Patagornis marshi* Moreno and Mercerat, 1891 [18] was chosen because of the near completeness of available materials. The largest South American bird, *B. burmeisteri*, whose affinities may lie with Phorusrhacidae or Galloanseres [19,20,23,56,57], was also included, but we restricted scoring of characters to the lectotype specimens (incomplete femur, tibiotarsus and tarsometatarsus) and a referred tarsometatarsus (FM-P13259); other listed material [19] is not robustly referred to this taxon [22] (see Discussion). Thus, a total of 48 taxa including seven outgroup palaeognaths were included in the analyses.

Size data were obtained for living taxa from Dunning [58]. For extinct taxa, body mass was estimated using measurements (see the electronic supplementary material). For analyses, all size data were logged to base 10.

The diet, and flight capabilities, of living taxa was obtained for waterfowl from Marchant & Higgins [59] and Kear [60], for galliforms, other neoavian taxa and palaeognaths from del Hoyo *et al.* [61], and for extinct *Dinornis* from Worthy & Holdaway [62]. Diet was classified into four states: 0, herbivorous; 1, mostly herbivorous, minor animal component; 2, omnivorous; 3, zoophagous animal predator, including insects/invertebrates. This trait was treated as a morphocline (ordered).

### 2.4. Phylogenetic analysis and ancestral state reconstruction

#### 2.4.1. Molecular backbone

Molecular data can often robustly retrieve relationships among modern (living and subfossil) taxa which are radically different from those based on only morphological data and in doing so can greatly influence the position of fossil forms [63]. Hence, the Bayesian and parsimony analyses were performed with and without enforcing a molecular backbone to provide objective assessments of phenotypic evolution [64]. The following studies were used to construct the molecular backbone: Aves and Neoaves [1,65,66], Palaeognathae [36,37], Galliformes [67,68] and Anseriformes [69–71]. The interspecific and higher relationships of all extant species and *Dinornis robustus* were enforced with this backbone constraint, so the only nodes not constrained in these analyses are those relating to fossil taxa. This backbone is included and implemented in the executable files in the electronic supplementary material.

#### 2.4.2. Parsimony analyses

Parsimony analyses of the morphological data matrix were performed using PAUP_4.0b10 [72], using heuristic searches with TBR branch swapping and 1000 random addition replicates per search. Inapplicable characters (coded as gaps ‘-’) were treated as missing data. Strict consensus trees were computed from the set of most parsimonious trees (MPTs), and clade support was assessed by bootstrapping [73] using the same settings and 1000 replicates. To prevent the bootstrap analyses from getting stuck on replicates with huge numbers of equally parsimonious trees, nchuck was set to 2000. The tree was rooted between extant palaeognaths and extant neognaths (see electronic supplementary material for nexus file).

Parsimony analyses were initially performed without any constraints (i.e. no molecular backbone), which resulted in topologies robustly contradicted by molecular data, and which raised suspicions of homoplasy artefacts (e.g. large-bodied taxa clustering together despite being morphologically dissimilar and/or geographically remote). To examine what may be driving the discordance, we conducted analyses with selective weighting of characters in unconstrained analyses, which showed that those of pectoral girdle elements (linked to loss of flight) were homoplasious and driving the contradictory topologies. This resulted in improved congruence with molecular analyses, supporting the view that the morphological data were extensively affected by homoplasy; the improved congruence also increased confidence in the molecular topology. As the primary aim of the analyses was to ascertain the relationships of fossil taxa in the context of the most likely phylogenetic relationships of extant taxa, we focused on the parsimony analyses with a molecular backbone. We examined the effect of the poorly known *Brontornis* by deleting this taxon and repeating the analysis. Also, because parsimony analyses robustly retrieved flightlessness as primitive for palaeognaths (an inference likely erroneous as abundant molecular data show that several ratite lineages each derive from a volant ancestor [37,39]), we repeated the analysis removing the ratite taxa to preclude their flightless condition causing problems with the optimization of a flightless ancestor for neornithines.

Diet, flight and body size were optimized on the primary trees in the ‘backboned’ analysis using parsimony via Mesquite [74].

Body mass was optimized on the MPT(s) using linear parsimony in Mesquite [74]; discrete phenotypic traits were optimized using parsimony (ordered or unordered as applicable) using PAUP.

#### 2.4.3. Bayesian inference

Bayesian analyses co-estimate topologies, branch lengths (anagenetic and chronological), ancestral states, divergence dates and evolutionary rates (see Lee & Palci [64] for a review of the benefits of this approach). The morphological, body size and ecological data were simultaneously analysed using tip-dated approaches that employ the ages of the fossil taxa [75,76], as implemented in BEAST 1.8.4 [77]. The root age was constrained to be broadly consistent with Prum *et al*. ([3]: analyses with *Vegavis* included). No other node age constraints were imposed, and the retrieved dates are generated from the phenotypic and stratigraphic information (geological age) contained in the fossil taxa (tips). Bayes Factors were used to test the need to accommodate rate variability among-characters (i.e. γ parameter) and among-lineages (i.e. relaxed clock). Each Bayesian analysis was repeated four times to confirm stationarity, with the post-burnin samples of all four runs combined for statistical analyses and consensus trees.

Bayesian phylogenetic analyses were performed with, and without, the molecular backbone. This is the first study to implement backbone constraints in BEAST 1; the relevant code is annotated in the xml file in the electronic supplementary material. In addition to estimating tree topology, divergence dates and morphological evolutionary rates, the Bayesian analyses also simultaneously estimated ancestral states for body size, diet and flight ability at each ancestral node, which can then be summarized on the timetree. Diet in fossil taxa was coded conservatively as ‘?’ and thus inferred using character optimization; the only exception was moa where gut contents indicate herbivory [62]. Our discussion focuses on the backboned analysis, as the analysis without the molecular backbone resulted in relationships among living taxa that are contradicted by a large body of genomic work.

## 3. Results

### 3.1. Analysis 1: parsimony analysis, no weighting and no backbone constraints

**Figure 1.**
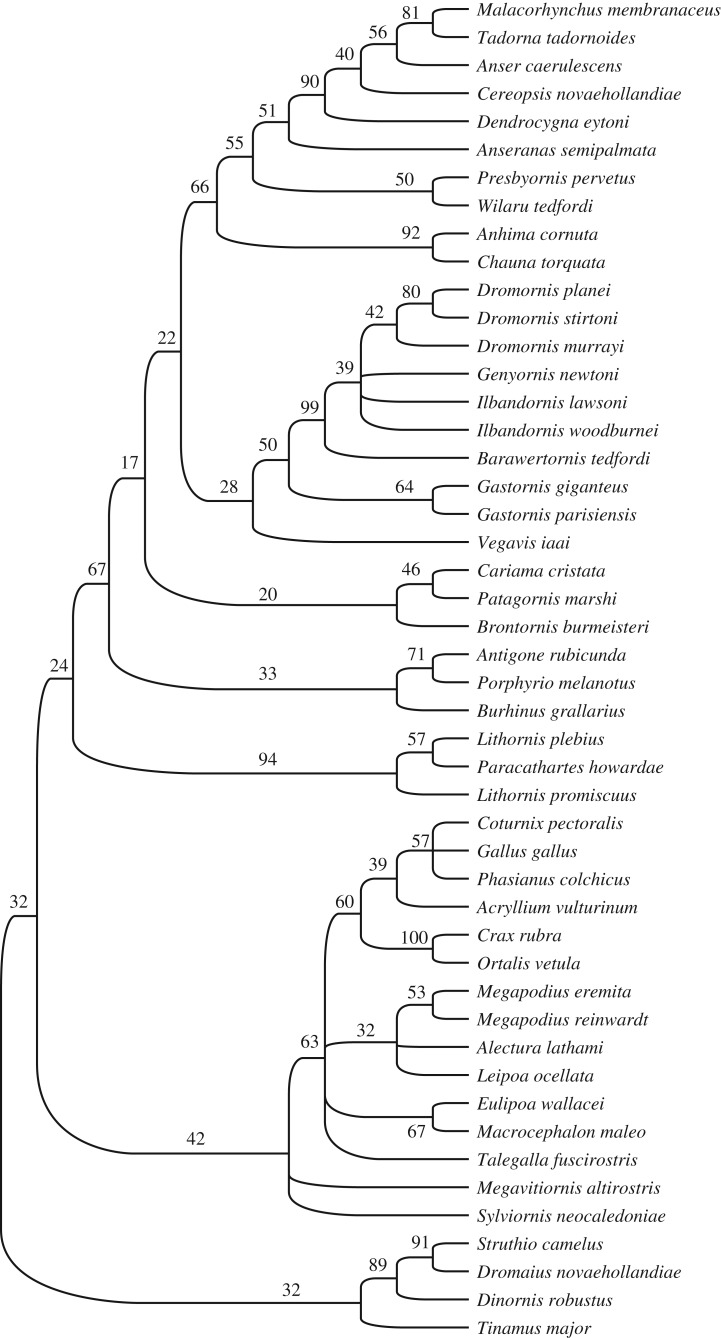
The consensus tree from Analysis 1 (no weights, no constraints); 15 MPTs, length 1648, CI = 0.2476, HI = 0.7524, RI = 0.6541 were found. Bootstrap support is indicated at nodes. Note that the tree could not be rooted in a way consistent with monophyly of the designated outgroup, Palaeognathae (tinamou, ratites and lithornithids).

An initial parsimony analysis with no character weighting and no constraints found 15 MPTs, tree length 1648, for which the consensus tree was reasonably well resolved (figure 1). Strong support (bootstrap support greater than 50%) for some major clades was found, e.g. for the Anseriformes (66%), Galliformes (63%) and Dromornithidae (99%). Relationships of the fossil taxa were identified as follows: the pairing of gastornithids and dromornithids received weak support (50%); *Wilaru* and *Presbyornis* were weakly supported (50%) as sister taxa, and their clade fell within crown-group anseriforms as the sister group of Anatoidea (Anseranatidae + Anatidae).

However, as Worthy *et al*. [17] found in a similar analysis, the more basal relationships were not consistent with many robustly supported molecular relationships. The tree could not be rooted so that palaeognaths formed a clade, while Galloanseres were also not monophyletic, in contrast to all recent analyses based on large molecular datasets (e.g. [1–3]). Instead, lithornithids fell on the stem of Anseriformes and distant from ratites and, in some trees, tinamous grouped with galliforms. Moreover, the neoavian taxa formed successive clades on the stem of anseriforms. This result was obtained despite our use of all characters previously identified as supporting these clades, and supports the view of Ericson [78] that osteological support for Galloanseres is weak, and in part reveals why taxonomists did not generally embrace the concept of Galloanseres until after the advent of molecular data (e.g. [79]). We consider it likely that our result was obtained in part because of our dense sampling of taxa within Galloanseres, especially of the less derived taxa such as megapodes, and use of outgroup taxa that are similar to galliforms (lithornithids and tinamou) reveals more homoplasy, which is not evident in smaller taxon sets. Furthermore, we consider that there is extensive homoplasy in the present dataset due to the combined effects of convergence arising from (i) reduced to lost wings related to flightlessness and (ii) enlarged pelvic girdles and legs due to large size.

### 3.2. Analysis 2: parsimony analysis with characters weighted and no backbone constraints

**Figure 2.**
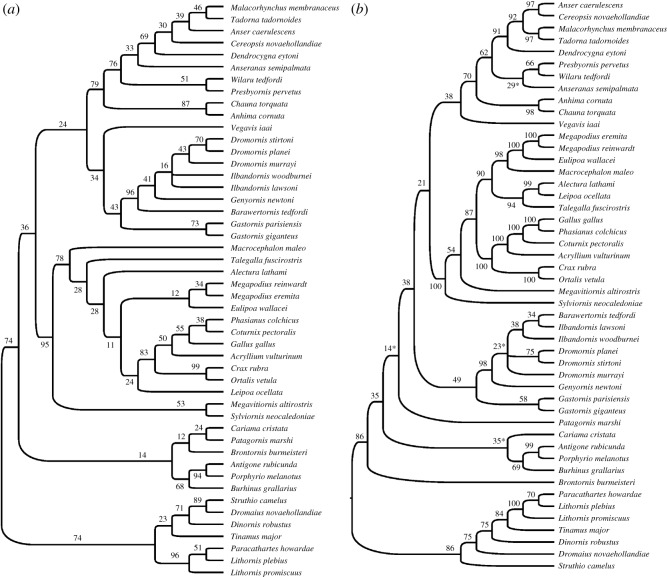
(*a*) The strict consensus tree from two MPTs found in Analysis 2 (characters weighted, no backbone constraint). (*b*) The strict consensus tree found in Analysis 3 (unweighted, molecular-based backbone constraint). In both (*a*) and (*b*) numbers are bootstrap support, and fossil taxa were free to move as dictated by the morphological data.

To counter the effects of homoplasy, which was evident in the unconstrained and unweighted parsimony analysis, an analysis was employed where, without topological constraints, characters were weighted such that cranial ones were emphasized (weight = 2.0), and all pectoral elements were down-weighted (0.5), relative to the remainder. This means that cranial elements gained most importance, and the pectoral girdle elements, which are expected to convergently degenerate in multiple flightless lineages, are given least importance in the analyses. The resultant strict consensus tree from two MPTs (length 1696.5, CI = 0.2564, HI = 0.7436, RI = 0.6711) had a topology (figure 2*a*) consistent with trees from large-scale molecular analyses. Galloanseres were monophyletic and formed the sister group to Neoaves, and these clades together formed the sister group to the outgroup palaeognaths, that was itself monophyletic. Lithornithids were strongly supported (74% bootstrap) as the sister group to remaining palaeognaths. Sylviornithids were strongly supported (95% bootstrap) as the sister group to galliforms. Gastornithids and dromornithids formed a clade (43% bootstrap) that was the weakly supported sister group to *Vegavis*, and these taxa were in turn weakly supported as the sister group of anseriforms. *Brontornis* resolved as sister to Cariamiformes, although with weak support.

Analysis 2 suggested that homoplasy relating to reduction in the pectoral girdles had a major impact on analyses when weighting was not invoked. To investigate this further, analyses (not shown) where only crania were weighted (2.0), or only pectoral girdle elements were down-weighted (0.5), resulted in neoavian taxa, respectively, making Galloanseres paraphyletic, or being the sister taxon of Galloanseres, revealed that homoplasy relating to the reduction of the pectoral girdle was a major issue. These analyses reveal that the morphological dataset contains substantial homoplasy, while the improved congruence (when weighting was invoked) also increases confidence in the molecular topologies.

### 3.3. Analysis 3: parsimony analysis with characters unweighted, topology constrained to molecular backbone

As the aim of these analyses was to assess the phylogenetic relationships of the various fossil taxa, and given the demonstrated issues of homoplasy, for Analysis 3 we enforced a backbone constraint of the relationships supported by genetic data, for all the extant taxa and *Dinornis* (as did Worthy *et al*. [17]), see §2.4.1. We left characters unweighted, allowing the pectoral characters to fully inform relationships of fossil taxa, which is important for those where wings were unreduced, e.g. *Vegavis* and presbyornithids. The fossils (dromornithids, gastornithids, *V. iaai*, presbyornithids, *S. neocaledoniae*, *M. altirostris*, *B. burmeisteri*, *P. marshi* and the three lithornithid species) were free to move within this backbone to their optimal positions as supported by morphology. This approach ensured that the fossil taxa were placed within a phylogenetic framework of living taxa which was robustly supported by (often large) amounts of available molecular evidence. A well-resolved tree, with a topology similar to that from Analysis 2, was recovered (figure 2*b*) (two MPTs, tree length = 1685, CI = 0.2421, HI = 0.7529, RI = 0.6438). The main difference is that the two fossils *Patagornis* and *Brontornis* separated from Neoaves and independently joining the stem of Galloanseres in poorly resolved positions. The dromornithid + gastornithid clade moved to a position as the sister taxon to galliforms + anseriforms, leaving *Vegavis* on the anseriform stem. Importantly, as in Analysis 2, there was no strong attraction of the large flightless non-palaeognaths to the ratites. An apomorphy list for this analysis is provided in the electronic supplementary material: Worthy et al Gallo 48taxa_290_PAUP_apolist_log.txt.

Analysis 3 reveals the instability of *Patagornis* and *Brontornis* which took an essentially unresolved position around the base of Neoaves (figure 2*b*), relationships which are almost certainly artefactual for the following reason. Because of the deeply nested position of lithornithids as the sister taxon to *Tinamus*, flightlessness was reconstructed as ancestral for palaeognaths, contradicting a large amount of genomic and biogeographical evidence [36,37,39]. As palaeognaths are the sister group to neognaths, this led to flightlessness also being reconstructed as ancestral for neognaths and Aves in general, which may cause large flightless forms (dromornithids, *Patagornis* and *Brontornis*) to be ‘pulled’ towards the base of the tree (figure 2*b*). Moreover, it has been suggested that missing data—most prevalent in *Brontornis*—can also pull taxa towards more basal phylogenetic positions [80].

### 3.4. Analysis 4: parsimony analysis with characters unweighted, topology constrained to molecular backbone, *Brontornis* removed

To investigate the impact of the large amount of missing data for *Brontornis* (73% characters unknown or inapplicable), an unweighted, constrained analysis was done where this taxon was excluded. A strict consensus of four MPTs, tree length = 1668, CI = 0.2446, HI = 0.7554, RI = 0.6457, revealed increased bootstrap support for the basal nodes: palaeognaths (86–99%), Gastornithiformes (49–64%) and Galloanseres (38–60%), the latter forming an unresolved polytomy of four clades (*Vegavis*, Anseriformes, Galliformes and Gastornithiformes). Also, *Patagornis*, in the absence of *Brontornis*, rejoins Neoaves as a cariamiform, a position highly consistent with biogeography and morphology; together, these observations show that the considerable missing data in *Brontornis* (the most incomplete taxon in the analysis) were causing homoplasy affecting these basal nodes.

### 3.5. Analysis 5: parsimony analyses with characters unweighted, topology constrained to molecular backbone, ratites removed

**Figure 3.**
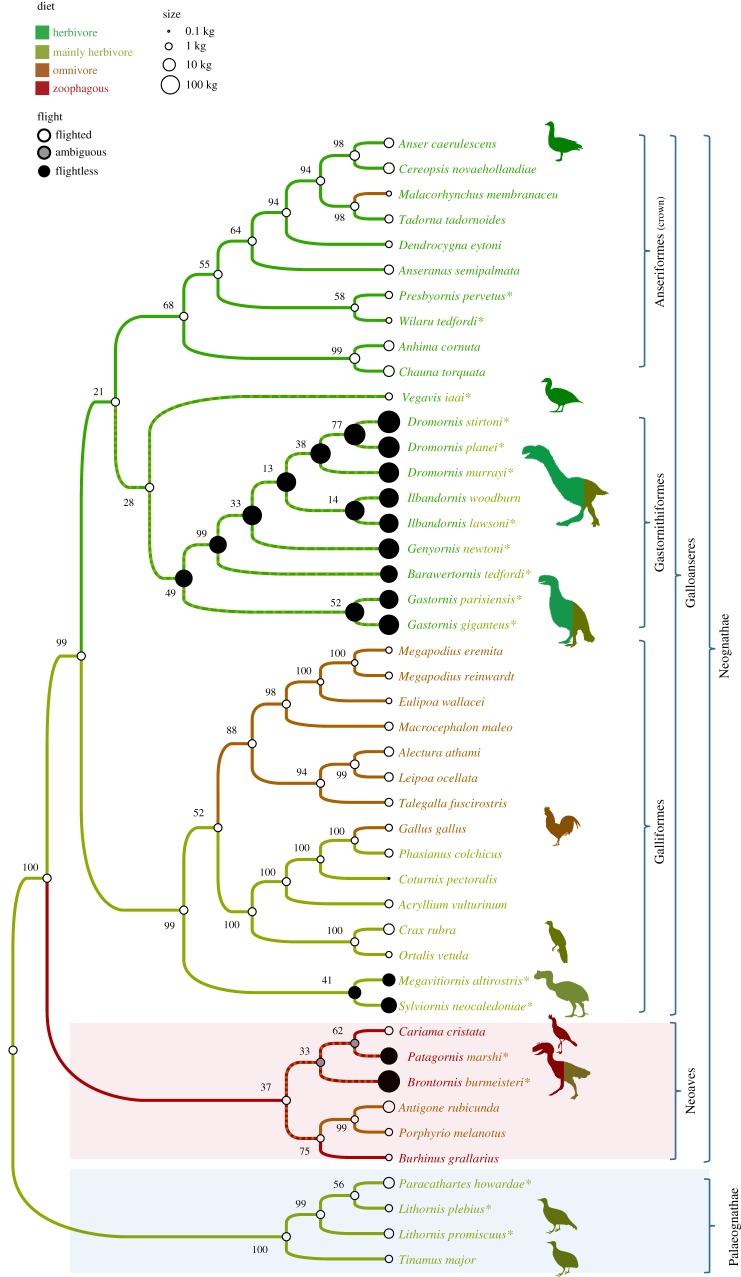
The parsimony tree (single best tree, length 1541, CI = 0.2596, HI = 0.7404, RI = 0.6519) found in the unweighted parsimony analyses with ratites excluded. Body size is shown by circle size, flight ability by circle shading and diet is indicated by branch colour (multiple colours = equally parsimonious). Numbers at nodes show bootstrap support. A molecular backbone was implemented for living taxa (and moa); fossil taxa were free to move as data dictated. Silhouettes from phylopic.org, individual artist credits in electronic supplementary material.

To investigate further the heterodox relationships found in Analysis 3, which improbably implies flightlessness in all basal avian nodes, we conducted parsimony analyses with the molecular backbone enforced, but with ratites excluded, and found a single tree of length 1541 (figure 3). As discussed above under Analysis 3, there is strong evidence that ratites do not reflect the ancestral palaeognath condition, which was most likely flighted and relatively small. Relationships of the fossil taxa were broadly similar to the unconstrained weighted Analysis 2 (figure 2*a*), with a dromornithid-*Gastornis* clade, and *Sylviornis* and *Megavitiornis* being stem galliforms, *Wilaru* and *Presbyornis* forming a clade within crown anseriforms and *Brontornis* grouping with the well-supported Cariamiformes (*Cariama* and *Patagornis*) among other neoavians. However, *Vegavis* is here united, albeit with weak support, with the dromornithid-*Gastornis* clade (and thus remains on the anseriform stem), and lithornithids are sister to tinamous. Support for basal nodes has improved markedly from that in Analysis 2, with Neognathae increasing from 74% to 100% and Galloanseres from 36% to 99%. Many nodes are poorly supported, but support for some of these increases greatly with the deletion of the poorly known *Brontornis*, as per Analysis 4. The apomorphy list for this analysis is given in the electronic supplementary material: Worthy et al Gallo45_ratites_removed_290_PAUP_apolist_log.txt. Corroborating Ericson's [78] contention that morphological support for Galloanseres is limited, we found only one unique synapomorphy supporting the group, i.e. char. 60, mandible with two cotylae CI = 1.000, 0 ⇒ 1. Only one other unambiguous character appears important: char. 28, basipterygoid processes located anterior to the basitemporal platform on the rostrum CI = 0.667, 0 ⇒ 1 (a reversal in *Gastornis* precludes CI = 1.000).

We used this analysis (which is also consistent with Analyses 2 and 4) to reconstruct the diet, volancy and body size of the fossil taxa (figure 3). All fossil taxa were reconstructed to have had a herbivorous or largely herbivorous diet, except for *Patagornis* and *Brontornis* (omnivorous or zoophagous equally parsimonious). Basal avian nodes (Aves, Palaeognathae, Neognathae, Galloanseres and Neoaves) are all reconstructed as flighted and relatively small (2.5–3.2 kg).

### 3.6 Bayesian inference

Bayes Factors favoured a model with evolutionary rate variability both among-characters (BF approx. 210) and among-lineages (BF approx. 123), and hence analyses employed the γ parameter and a relaxed (UCLN) clock.

**Figure 4.**
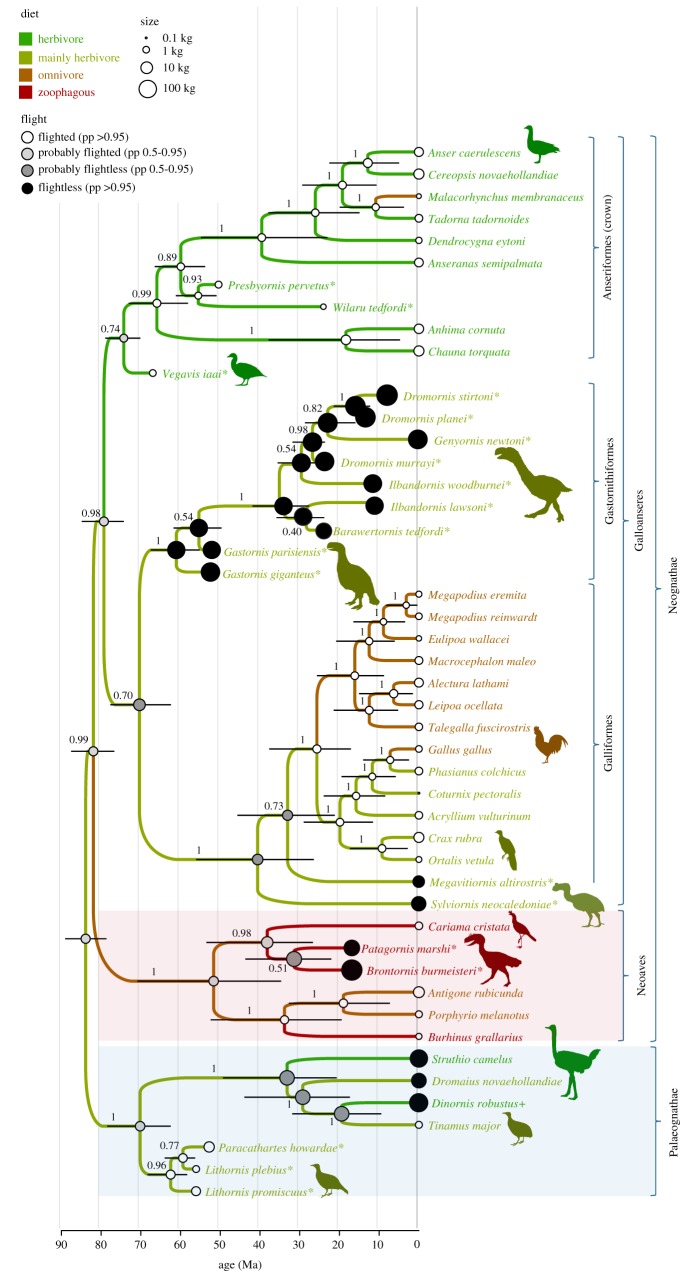
The Bayesian tree (maximum clade credibility consensus) from 3200 post-burnin sampled trees from the tip-dated Bayesian analysis showing reconstructed diet, body size and divergence ages of clades. Body mass is shown by circle size, flight ability by circle shading and diet is indicated by branch colour. Numbers at nodes show posterior probabilities. Divergence dates are indicated by the scale below the tree with confidence intervals shown as bars at nodes. A molecular backbone was implemented for living taxa (and moa); fossil taxa were free to move as data dictated. Silhouettes from phylopic.org, individual artist credits in electronic supplementary material.

The tip-dated Bayesian analysis employing a molecular backbone is summarized in figure 4. Dromornithids and *Gastornis* form a robust clade (posterior probability (pp) = 1.0), basal on the galliform stem, followed by *Sylviornis* and *Megavitiornis*. All these taxa are robustly excluded (pp = 1.0) from crown galliforms. *Brontornis* and *Patagornis* are robustly united (pp = 0.99) with seriema (*Cariama*) within Neoaves. The relationships of the controversial flighted forms are also well resolved. *Wilaru* and *Presbyornis* are strongly united (pp = 0.94) and form the sister group to Anatoidea (Anseranatidae + Anatidae) (pp = 0.90). Notably, *Vegavis* is on the anseriform stem and robustly excluded (pp = 0.99) from the anseriform crown; lithornithids are basal palaeognaths (pp = 1.0) and robustly excluded from the palaeognath crown (pp = 1.0). Divergence dates for basal avian branches (figure 4) are broadly consistent with previous work and imply the four ordinal-level clades of Galloanseres originated in the Late Cretaceous, but divergences within crown palaeognaths and crown galliforms are very shallow (see §4). The character support for major clades and the complete apomorphy list for this analysis are given in the electronic supplementary material (see the Beast apomorphy list). Support for Galloanseres is relatively weak, consisting of the same characters as identified in parsimony analyses.

All fossil taxa are reconstructed with a herbivorous or largely herbivorous diet, except for *Patagornis* (zoophagous) and *Brontornis* (zoophagous). Basal avian nodes (Aves, Palaeognathae, Neognathae, Galloanseres and Neoaves) are reconstructed as flighted and relatively small (2.5–3.6 kg). However, nodes between crown palaeognaths and crown galliforms are reconstructed as probably flightless, which is unlikely (see §4).

## 4. Discussion

### 4.1. Relationships of fossil Galloanseres

In a series of parsimony analyses, we have demonstrated that: (i) parsimony analyses of unweighted data without molecular backbone constraints resulted in highly heterodox relationships among major neornithine clades; (ii) selective weighting of characters identifies extensive homoplasy, relating to reduced pectoral girdle and wing elements, as the cause of most of the discordance between morphological and molecular phylogenies; (iii) use of a molecular backbone enables the fossil taxa to be analysed in the context of a phylogenetic framework that most likely approaches the true evolutionary relationships and also enables full consideration of the character signal for all skeletal parts; (iv) missing data are problematic and compromise perceived relationships for some taxa, e.g. *Brontornis*; and (v) parsimony is unable to reconstruct plausible ancestral states for flight in palaeognaths because of convergent loss of flight and the attraction of tinamous and lithornithids.

In our focal analyses, we employed a molecular backbone that constrained the relationships of the extant taxa and *Dinornis* to the relationships that are robustly inferred from extensive molecular data (see §2.4.1). The fossil taxa were free to associate as dictated by the morphological data. This tree was very similar to that obtained when characters were weighted to ameliorate homoplasy, and when problematic taxa such as ratites and *Brontornis* were deleted. Bayesian methods which simultaneously consider morphological data and stratigraphic ages also retrieved a tree topology very similar to the preferred parsimony analysis.

The focal parsimony and Bayesian analyses (figures 3 and 4) found strong support for the Galloanseres (bootstrap = 99%, pp = 0.98) comprising four distinct clades (Anseriformes, Galliformes, Gastornithidae + Dromornithidae and *Vegavis*) whose interrelationships are weakly resolved, but which each originated in the Late Cretaceous, as inferred by Livezey [44], rather earlier than hypothesized by both Jarvis *et al*. [1] and Prum *et al*. [3]. The poor resolution of these four clades stems mainly from the gastornithid–dromornithid clade being attracted to both the stem of Galliformes (figure 4) and the stem of Anseriformes (figure 3). A Gondwanan origin for Galloanseres [44] is supported by the observation that all clades either have their basal members distributed on Gondwanan fragments (Galliformes, Anseriformes) or lived on Gondwana (*Vegavis*, Dromornithidae). The giant extinct Australian mihirungs (Dromornithidae) are robustly united in a clade (bootstrap = 49%; pp = 1.00) with the similarly large Northern Hemisphere Gastornithidae. Resolution within Dromornithidae is low primarily because of missing data, partly because all species have markedly reduced pectoral elements, but also because skull elements are highly fragmentary or unknown in four of the seven taxa.

Notably, *Gastornis* is paraphyletic in the Bayesian analysis, with *G. parisiensis* alone being the sister of dromornithids. This arrangement may relate to missing data in *G. parisiensis*, but there are minimally 20 coded character differences between *G. parisiensis* and *G. giganteus*. Some of these appear important as they are often conservative within genera, for example: char. 54, foramen pneumaticum basiorbitale in quadrate present in *G. parisiensis* (absent in *G. giganteus*); char. 93, coracoid with foramen nervi supracoracoidei (absent); char. 189, femur lacking fossa trochanterica (present); char. 194, femur impressiones obturatoriae bulbous proximally and no large prominences on caudolateral margin more distally (impressiones obturatoriae prominent proximally and a distinct prominence more distally on caudolateral margin for m. ischiofemoralis); char. 219, femur trochlea fibularis short and merges with side of trochlea lateralis proximal to its distal end (long, equal extent with trochlea lateralis); char. 248 tibiotarsus with distal opening of canalis extensorius aligned transversely on shaft like galliforms (across shaft as in anseriforms); char. 257, tarsometatarsus hypotarsus distinctly narrower than half proximal width (approximately half); char. 262, sulcus extensorius deep and well defined at midlength tarsometatarsus (shallow); char. 263, tuberositas m. tibialis cranialis on tarsometatarsus with two distinct tuberosities (one); and char. 271, fossa metatarsi I absent or obsolete (present well marked). These differences between the two *Gastornis* species are doubtless driving their potential non-monophyly and raise the question as to whether the two species should be considered congeneric. The differences raise the possibility that some of the similarities in these species of *Gastornis* are homoplasic and related to convergent evolution of gigantism and slow walking habit, and that *contra* recent consensus (e.g. [14,81]), these taxa may be generically distinct, as concluded by Martin [13]. However, we recognize that a full assessment of this issue is beyond the scope of this project and merely flag the possibility. If the two species of *Gastornis* indeed form an evolutionary grade, then separate origins from a smaller volant ancestral lineage are probable, in a similar manner to that now invoked for ratites among crown palaeognaths [37,39].

A parsimonious interpretation of the dromornithid-*Gastornis* clade might conclude that the common ancestor of this group was large and flightless, but this appears biologically implausible, given their Paleocene age and wide geographical distribution. Rather, their history was most likely very similar to that now recognized for living flightless palaeognaths (ratites). If tinamous were no longer extant, it would not be possible to use DNA to establish their nested position within ratites (e.g. [36]) and, therefore, analyses would conclude that ratites were monophyletic with a common ancestor that was large and flightless, the conclusion reached before molecular evidence was analysed (e.g. [82]). It is now well established from abundant molecular data that volant ancestors repeatedly gave rise to parallel lineages of giant flightless ratites (e.g. [36,37]). In a similar manner, we suspect that a small volant stem-galloansere taxon spawned both the various species of *Gastornis* and the dromornithids following Early Paleogene dispersal to the areas those clades inhabited.

The gastornithid–dromornithid clade can be formally recognized as Gastornithiformes Stejneger, 1885 [83]. In the Bayesian analyses, gastornithiforms are weakly resolved as the sister group to galliforms (figure 4): in the parsimony analyses, they are weakly resolved as the sister group to *Vegavis* and then to Anseriformes (figure 3). Resolution of this issue will likely require discovery of new relevant fossils, especially cranial material of *Vegavis* or a near relative.

*Vegavis iaai* is strongly supported as a member of Galloanseres, but its sister group (Gastornithiformes or Anseriformes) varied across analyses, despite having near 50% of data coded. However, it is consistently and robustly excluded from crown group Anseriformes, with a parsimony bootstrap of approximately 68% and a Bayesian pp of 0.99 (cf. [40,42]). This has significance because of the widespread use of *Vegavis* in calibrating molecular analyses: *Vegavis* and its geological age of 69–66 Ma [42] should, in future, be used only as the minimum date for Galloanseres as a whole, not for Anseriformes or any more restricted clade such as Anatoidea, *contra* Clarke *et al*. [40,42]. This change will reduce molecular clock divergence dates, perhaps substantially. This position also supports differentiation at the family level for *Vegavis*: as it lies outside of Anseriformes, it should have an equivalent ranking (table 1).

**Table 1.** An amended classification of Galloanseres, listing only taxa discussed herein, developed from Dickinson & Remsen [47].

infraclass neognathae pycraft, 1900 [84]
parvclass galloanseres sibley and ahlquist, 1990 [79]
order gastornithiformes stejneger, 1885 [83]
suborder gastornithes stejneger, 1885 [83]
gastornithidae fürbringer, 1888 [85]
suborder dromornithes fürbringer, 1888 [85]
family dromornithidae fürbringer, 1888 [85]
order galliformes (Temminck, 1820) [86]
suborder sylviornithes new taxon
family sylviornithidae mourer-chauviré and balouet, 2005 [87]
suborder galli wetmore 1960 [29]
family megapodiidae lesson, 1831 [88]
family cracidae rafinesque, C.S. 1815 [89]
family numididae de selys longchamps, 1842 [90]
family odontophoridae gould, 1844 [91]
family phasianidae horsfield, 1821 [92]
order anseriformes (Wagler, 1831) [93]
suborder anhimae wetmore and miller, 1926 [94]
family anhimidae stejneger, 1885 [83]
suborder anseres wagler, 1831 [93]
superfamily presbyornithoidea wetmore, 1926 [55]
family presbyornithidae wetmore, 1926 [55]
superfamily anatoidea (leach, 1819) [95]
family anseranatidae sclater, 1880 [96]
family anatidae leach, 1819 [95]
order vegaviiformes new taxon
family vegaviidae agnolin *et al*. [97]
genus *vegavis* clarke *et al*. [40]

Presbyornithids were found to be strongly supported as crown Anseriformes as a sister group to Anatoidea (i.e. anseriforms other than anhimids), rather than as a sister to Anatidae as previously hypothesized [43,44]. *Wilaru* and *Presbyornis* formed a clade in all analyses, as concluded by De Pietri *et al*. [53], although support was limited by the lack of skull material for *W. tedfordi* and resultant missing data. Notably, there was no support for a *Burhinus*–*Wilaru* pairing as the original classification of *W. tedfordi* would predict [52]. The dual hemispheric distribution of presbyornithids [53] indicates a near global dispersal of this group by the Early Paleogene. By contrast to browsing gastornithiforms, their specialized filter-feeding adaptations for living around lakes apparently required flight to access suitable habitat, and so all presbyornithids remained volant. This group survived longest in Australia along with several other basal anseriform lineages [53].

In all analyses, *Sylviornis* and *Megavitiornis* are found strongly supported either as a grade or a clade of stem Galliformes. However, support for them being a grade is low (pp = 0.65, bootstrap = 51%), and it is possible that this is an artefact of missing data, which is greatest in *Megavitiornis*. Their geographical proximity also does not preclude a common flightless ancestor. Hence, we do not alter the composition of Sylviornithidae (*sensu* [17]) and here propose extending the definition of Galliformes to include Sylviornithidae recognized at the subordinal level.

### 4.2. Relationships of lithornithids

While the main aim of the project was to determine the relationships of the fossil Galloanseres, inclusion of three volant Paleogene lithornithids allowed a broad assessment of the relationships of this group. It has been well accepted that lithornithids are palaeognaths since Houde [46], but their position within palaeognaths is controversial [35,46]. Tinamous, the only extant volant palaeognaths, were once considered close to galliforms (e.g. [85,98]), but, as first identified by Parker [99] and later reinforced by the seminal work of Pycraft [84], they have been considered to be palaeognaths (e.g. [1,2,32,34,37,65,82,100–104]), but see Houde [46].

Tinamous and lithornithids are both volant, chicken-sized taxa, that have long been known to share many osteological features [46]. Our new analyses of lithornithids confirm that there is a strong signal for lithornithids being the sister taxon of either tinamous [17,33,46,103,105] or all extant palaeognaths [34,35,39]. In the tip-dating Bayesian analysis (figure 4), lithornithids are recovered as stem palaeognaths with pp = 1. This is consistent with their early age and results in flight and intermediate body size being reconstructed as primitive for palaeognaths and other basal avian nodes, a scenario with strong molecular support (e.g. [37,39]). Weighted parsimony analyses also recovered lithornithids in this basal palaeognath position (figure 2*a*), but molecular backbone parsimony analyses united them with tinamous and thus nested within palaeognaths (figure 2*b*), which is inconsistent with their age [39], and also result in flightlessness and large body size being reconstructed for palaeognaths and many basal avian nodes. Thus, evolutionary considerations favour the basal position of lithornithids and suggest that tip-dated Bayesian methods can better overcome homoplasy issues bedevilling morphological analyses of palaeognaths (e.g. [32–34,36,37,39,104]).

### 4.3. Relationships of *Brontornis*

Our analyses for the relationships of *Brontornis* were compromised by missing data, in part because this is a rare, poorly known taxon, but also because we conservatively opted to use only robustly assigned material (i.e. specimens wherein preserved elements overlapped those present in the lectotype). Despite this, we found strong support for *Brontornis* being a member of Neoaves (figures 3 and 4). In the tip-dated Bayesian analysis (figure 4), *Brontornis* is a member of Neoaves, grouping with *Patagornis* and *Cariama* with pp = 1, and as the sister taxon to *Patagornis*, although with weak support. *Brontornis* shares an autapomorphic block-like structure of the hypotarsus (see below) with Phorusrhacidae and Cariamidae, see Alvarenga & Höfling ([19], figs 8 and 9). However, under parsimony with a molecular backbone (figure 3), we recover *Brontornis* as the sister taxon of Cariamiformes (*Cariama* + *Patagornis*), albeit with weak support, rather than as the sister group of *Patagornis* alone. It is possible that the abundant missing data (73%) resulted in *Brontornis* slipping towards the base of the clade, as found in models testing the effect of missing data on topological relationships [80], rather than it having an independent origin to phorusrhacids.

*Brontornis* has often been considered to be a neoavian related to Phorusrhacidae [19], although usually distinguished as the subfamily Brontornithinae. Moreno & Mercerat [18] stated that this bird was related to *Cygnus*, based on hind limb bone morphology. However, *Brontornis* was clearly separated from phorusrhacids by Dolgopol de Saez [106] and placed as the family Brontornithidae in the new order Brontornithes based on characters of the leg bones, including that the ungual phalanges were flattened, the pons supratendineus of the tibiotarsus was lacking and the canalis interosseus distalis was unbifurcated and opening only to the incisura intertrochlearis lateralis. Although these features are highly contrasting with Phorusrhacidae, they were not considered significant by most subsequent authors (e.g. [19]), and *Brontornis* was recognized only as distinct at the subfamily level. Support for its greater distinction grew following Agnolin's [20] explicit proposal that *B. burmeisteri* was a basal member of Anseriformes. Thereafter, *Brontornis* has been listed in Brontornithidae and as a member of Galloanseres by most commentators [21–23,57]. Recently, Buffetaut [24] has revived the significance of the features listed by Dolgopol de Saez [106] and again argued that Brontornithidae is justified.

**Figure 5.**
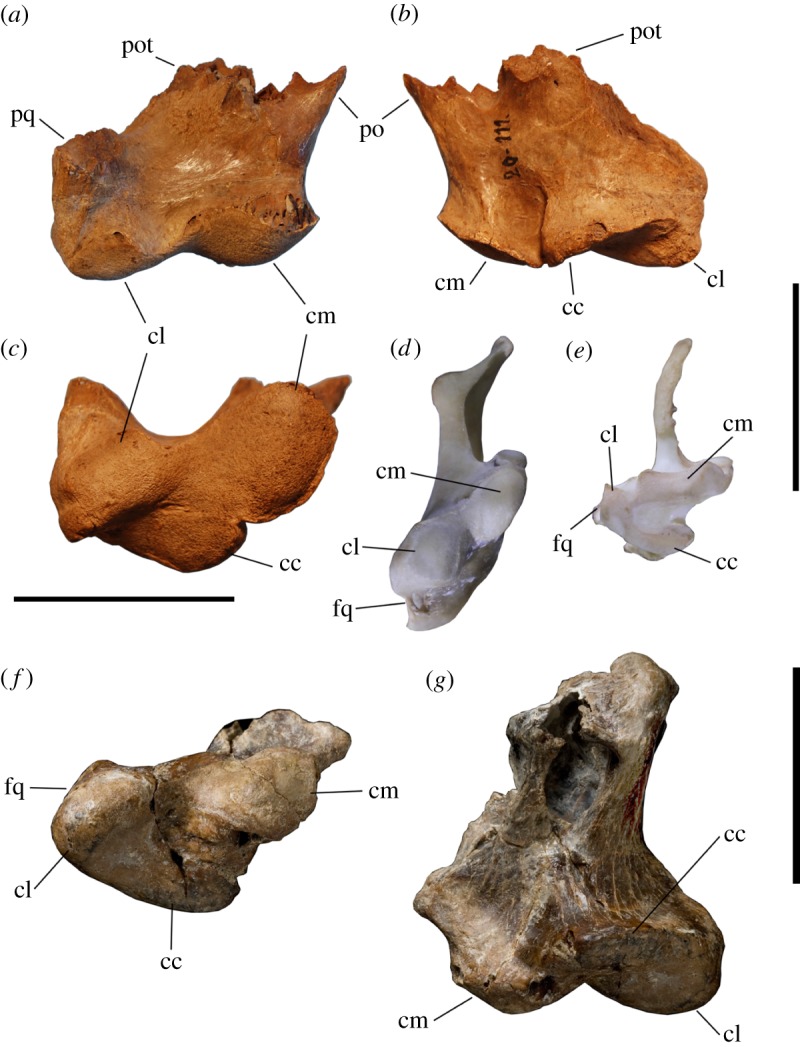
Right quadrates: (*a*–*c*) mandibular part MLP 20–111 referred to *B. burmeisteri*; (*d*) *Anseranas semipalmata* SAM B36790; (*e*) *Threskiornis spinicollis* SAM B48531; (*f*,*g*) *P. marshi* BMNH-A516; shown in (*a*) anterior view; (*b*,*g*) posterior and (*c–f*) ventral (anterior to top of figure) views. *Anseranas semipalmata* shows the typical galloansere condition of two condyles, markedly differing from the three-condylar state of Neoaves. cc, condylus caudalis; cl, condylus lateralis; cm, condylus medialis; fq, fovea quadratojugalis; po, ventral margin base of processus orbitalis; pot, base of processus oticus; pq, pars quadratojugalis of lateral process. Scale bars, (*a*--*c*) 50 mm; (*d*,*e*) 10 mm; (*f*,*g*) 25 mm.

However, it is noteworthy that Agnolin [20] based his interpretation primarily on the morphology of the quadrate (MLP 20–111). In Tambussi & Degrange [22, fig. 7.3], this quadrate is depicted in photographs, revealing that Agnolin [20] misinterpreted the bone by interpreting the anterior side as posterior and vice versa. Moreover, the distal view shown in Tambussi & Degrange [22, fig. 7.3] clearly shows the quadrate has three condyles, as shown here in figure 5 compared to *Patagornis*, with the condylus caudalis reduced and placed relatively more dorsal than the other two. A quadrate with three condyles means that this bone cannot be a galloansere, as members of this group have two condyles [107,108]. So, if this quadrate is correctly associated with *B. burmeisteri* (see [22]), then *Brontornis* is not of Galloanseres.

The features that Dolgopol de Saez [106] and Buffetaut [24] considered significant for distinguishing Brontornithidae may have little value in higher taxonomy as they are losses or reductions that have occurred in multiple lineages. The canalis interosseus distalis typically extends from the dorsal surface to the plantar surface of the tarsometatarsus as the foramen vasculare distale with a branch to an opening in the incisura intertrochlearis lateralis. The canalis is reduced or lost independently in many palaeognaths: in *Aepyornis*—is reduced to an intertrochlear notch; in *Dromaius* and *Casuarius—*is small; in *Struthio* and Dinornithiformes—is lost entirely [34]; and in *Apteryx—*it varies from present and small with both plantar and incisura openings, to occasionally lost entirely [109]. Similar losses occur in Galloanseres, such as in *Sylviornis* where it is markedly reduced plantarly [17], in dromornithids where the plantar branch is lost and the inter-trochlear branch is either markedly reduced or lost [7], or even lost entirely in some anatids, e.g. *Cnemiornis calcitrans*, see Worthy *et al*. [110].

Flattened ungual phalanges have also been attained independently in some ratites (e.g. in *Struthio*) and in some Galloanseres (e.g. all dromornithids), which have acquired short, flattened ungual phalanges. The absence of an ossified pons supratendineus on the tibiotarsus may be the ancestral state [13]. It is absent in lithornithids, *Struthio* and casuariids; however, a pons is present in the oldest galloansere *Vegavis* and is variably present in *Apteryx*, present in all species of Dinornithiformes and tinamous [34] and in most Neoaves, although with the notable exception of Strigiformes, suggesting multiple acquisitions of the derived state in Neornithes. Even among Cariamiformes, some taxa have lost the ossified pons supratendineus, such as Ameghinornithidae and *Salmila* [111,112]. Its presence in *Cariama* and in phorusrhacids and labile nature in palaeognaths raises the possibility of secondary loss in *Brontornis*.

In summary, loss or reduction of the canalis interosseus distalis and the unguals becoming shortened and flattened have occurred repeatedly in the evolution of massive terrestrial birds and seems therefore not a strong basis on which to establish major (e.g. familial) differences. While the presence or absence of an ossified pons supratendineus is consistent within large clades such as families, its absence does not negate a possible sister group relationship with a clade given parallel development of extreme large mass. Such features have parallels to those related to loss of flight such as markedly reduced wings and pectoral elements, which is not usually considered significant to denote major groups (e.g. variation within genera of rails and ducks, among species of *Porphyrio* or *Anas*; see [113,114]).

Undoubtedly, there are some similarities in the hind limb bones of *Brontornis* and Phorusrhacidae: the lateral excavation of the medial surface of the condylus lateralis of the femur and the block-like hypotarsus. However, this block-like hypotarsus is markedly different from the triangular-shaped (block-like) hypotarsus of phorusrhacids [23]. Symphysial shape and dorsoventrally deepened bill together with femur, tibiotarsus and tarsometatarsus shape and structure differ substantially from that of phorusrhacids (although this is highly probably correlated with the large body mass achieved by *Brontornis*).

We have shown conclusively that *Brontornis* is not a galloansere and shares an autapomorphic hypotarsal structure with Cariamiformes, supporting the original view that it is more closely related to phorusrhacids (e.g. [19,56]). Its extreme adaptations related to large body mass and associated simplification in tarsometatarsal morphology justify its distinction, although to confirm its discrimination as Brontornithinae in Phorusrhacidae, as advocated by Alvarenga & Höfling [19], requires much more complete material.

### 4.4. Phylogenetic constraints on dietary ecology of giant extinct birds

We conservatively treated diet as unknown in all fossil birds (except for *Dinornis*, where direct gut content remains have been recorded [62]). This allowed us to infer the diet of each fossil bird solely based on its phylogenetic position and compare this to inference from functional morphology. Both the parsimony and Bayesian (figures 3 and 4) analyses reveal major phylogenetic constraints in the diet of all giant extinct birds. Essentially, the broad diet of all giant flightless birds is dictated by the diets of their smaller flighted ancestors. The majority of extant Galloanseres are either herbivores or plant-dominated omnivores, although it is recognized that the Mergini (seaducks), a highly derived clade within Anatidae, are primarily zoophagous, being piscivorous [60]. Phylogenetically, gastornithids are thus robustly reconstructed as primarily herbivorous—adding support to recent morphological analyses that have argued these birds were herbivorous [11,12,81,115,116] in contrast to those who have advocated a zoophagous diet (e.g. [10,117]). Similarly, dromornithids are phylogenetically reconstructed as primarily herbivorous as is generally recognized (see [6] and references therein), in contrast to suggestions of zoophagy [118–120]. Sylviornithids were reconstructed to have a primarily herbivorous diet with a minor omnivorous component, similar to cracids or phasianids. The diet of *Wilaru* and *Presbyornis* was robustly reconstructed as primarily herbivorous. Again, this is consistent with independent predictions of a herbivorous filter-feeder from functional morphology [121]. Only in Neoaves is zoophagy widespread. Accordingly, the phorusrhacid *Patagornis* is recovered as zoophagous or omnivorous/zoophagous, consistent with existing views that it was a predator [19,122,123]. *Brontornis* is reconstructed as omnivorous or zoophagous (*sensu* [23,123]). Thus, despite up to 100-fold increases in body mass (figures 3 and 4), and massive morphological changes, the diets of gastornithids, dromornithids, sylviornithids, phorusrhacids and *Brontornis* have remained conservative, closely mirroring their respective small flighted ancestors.

## 5. Conclusion

Parsimony analyses, which correct for homoplasy and missing data, and tip-dated Bayesian methods, reveal broadly concordant relationships for extinct Galloanseres. Both approaches show that this group is monophyletic and comprises four major clades, the extant Galliformes, extant Anseriformes, extinct *Vegavis* and extinct Gastornithiformes. *Vegavis*, the only well-supported neornithine from the Cretaceous, is robustly excluded from crown Anseriformes, resulting in major implications for the use of this taxon in calibrations of molecular analyses. Presbyornithids are confirmed as anseriforms, but are the sister group to Anatoidea (i.e. anseriforms other than anhimids), rather than the sister group to Anatidae. The Australian *Wilaru tedfordi* is confirmed as a presbyornithid. Gastornithiformes includes Dromornithidae, which are thus not anseriforms, and this clade of giant flightless birds probably arose by independent dispersal of volant ancestors in a similar way as is widely recognized for ratite palaeognaths. South America's largest bird *Brontornis burmeisteri* is shown to be a neoavian taxon, not a galloansere, and most likely either a cariamiform or a closely related taxon. Our Bayesian analyses place lithornithids as a sister group to crown palaeognaths and thus suggest their morphology reflects that of ancestral palaeognaths and neornithines.

## Supplementary Material

Supplementary text Document

## Supplementary Material

A complete list of Morphological characters

## Supplementary Material

Supplementary text Document;A complete list of Morphological characters;Data on mass, diet and age of study taxa;3 Apomorphy lists as separate files;Executable files x2

## Supplementary Material

Supplementary text Document;A complete list of Morphological characters;Data on mass, diet and age of study taxa;3 Apomorphy lists as separate files;Executable files x2

## Supplementary Material

Supplementary text Document;A complete list of Morphological characters;Data on mass, diet and age of study taxa;3 Apomorphy lists as separate files;Executable files x2

## Supplementary Material

Supplementary text Document;A complete list of Morphological characters;Data on mass, diet and age of study taxa;3 Apomorphy lists as separate files;Executable files x2

## Supplementary Material

Supplementary text Document;A complete list of Morphological characters;Data on mass, diet and age of study taxa;3 Apomorphy lists as separate files;Executable files x2

## Supplementary Material

Data on mass, diet and age of study taxa

## Data accessibility

Additional resources are in the electronic supplementary material files linked to this article.
