# Correction to ‘The evolution of giant flightless birds and novel phylogenetic relationships for extinct fowl (Aves, Galloanseres)'

**DOI:** 10.1098/rsos.171621

**Published:** 2017-11-15

**Authors:** Trevor H. Worthy, Federico J. Degrange, Warren D. Handley, Michael S. Y. Lee

*R. Soc. open sci.* **4**, 170975. (Published 11 October 2017). (doi:10.1098/rsos.170975)

Table 1 was presented incorrectly in the published paper. The corrected table is shown below.

**Table 1.** An amended classification of Galloanseres, listing only taxa discussed herein, developed from Dickinson & Remsen [47].

Infraclass Neognathae Pycraft, 1900 [84]
Parvclass Galloanseres Sibley and Ahlquist, 1990 [79]
Order Gastornithiformes Stejneger, 1885 [83]
Suborder Gastornithes Stejneger, 1885 [83]
Family Gastornithidae Fürbringer, 1888 [85]
Suborder Dromornithes Fürbringer, 1888 [85]
Family Dromornithidae Fürbringer, 1888 [85]
Order Galliformes (Temminck, 1820) [86]
Suborder Sylviornithes new taxon
Family Sylviornithidae Mourer-Chauviré and Balouet, 2005 [87]
Suborder Galli Wetmore 1960 [29]
Family Megapodiidae Lesson, 1831 [88]
Family Cracidae Rafinesque, C.S. 1815 [89]
Family Numididae de Selys Longchamps, 1842 [90]
Family Odontophoridae Gould, 1844 [91]
Family Phasianidae Horsfield, 1821 [92]
Order Anseriformes (Wagler, 1831) [93]
Suborder Anhimae Wetmore and Miller, 1926 [94]
Family Anhimidae Stejneger, 1885 [83]
Suborder Anseres Wagler, 1831 [93]
Superfamily Presbyornithoidea Wetmore, 1926 [55]
Family Presbyornithidae Wetmore, 1926 [55]
Superfamily Anatoidea (Leach, 1819) [95]
Family Anseranatidae Sclater, 1880 [96]
Family Anatidae Leach, 1819 [95]
Order Vegaviiformes new taxon
Family Vegaviidae Agnolin *et al*. [97]
Genus *Vegavis* Clarke *et al*. [40]

