# Supplementary material for: The evolution of giant flightless birds and novel phylogenetic relationships for extinct fowl (Aves, Galloanseres)
Source: R Soc Open Sci. 2017 Oct 11;4(10):170975. doi: 10.1098/rsos.170975 (PMC5666277; doi:10.1098/rsos.170975)
Supplement: Supplementary text Document [file rsos170975supp1.docx]

**Supplementary Data:**

**The evolution of giant flightless birds and novel phylogenetic relationships for extinct fowl (Aves, Galloanseres)**

Trevor H. Worthy^a,*^, Federico J. Degrange^b^, Warren D.Handley^a^ and Michael S. Y. Lee^a,c^

^a^ School of Biological Sciences, Flinders University, Adelaide, South Australia, Australia. Email: [trevor.worthy@flinders.edu.au](mailto:trevor.worthy@flinders.edu.au); [mike.lee@flinders.edu.au](mailto:mike.lee@flinders.edu.au); [warren.handley@flinders.edu.au](mailto:warren.handley@flinders.edu.au);

^b^ Centro de Investigaciones en Ciencias de la Tierra (CICTERRA), UNC, CONICET, Av. Vélez Sársfield 1611, X5016GCA, Córdoba, Argentina. Email: [fjdino@gmail.com](mailto:fjdino@gmail.com);

^c^ Earth Sciences Section, South Australian Museum, North Terrace, Adelaide, South Australia, Australia. Email: [mike.lee@samuseum.sa.gov.au](mailto:mike.lee@samuseum.sa.gov.au).

*****Corresponding author. Email: [trevor.worthy@flinders.edu.au](mailto:trevor.worthy@flinders.edu.au) (THW)

**Abbreviations**

**AMNH**, American Museum of Natural History, New York, U.S.A.; **CIT-O**, Colección de Aves Actuales del CICTERRA, Córdoba, Argentina; **CORD**, Museo de Paleontología de la Universidad Nacional de Córdoba, Córdoba, Argentina; **CPC**, Commonwealth Palaeontological Collections, Bureau of Mineral Resources, Canberra, Australia (now Geoscience Australia); **FM**, Field Museum, Chicago, U.S.A.; **LACM**, Vertebrate Zoology, Natural History Museum of Los Angeles County, Los Angeles, California, U.S.A.; **MACN**, Museo Argentino de Ciencias Naturales, “Bernardino Rivadavia”, Buenos Aires, Argentina; **MLP**, Museo de La Plata, La Plata, Argentina; **MPM**, Museo Padre Molina, Río Gallegos, Santa Cruz, Argentina; **NHMUK**, Natural History Museum, London, United Kingdom; **NTM**, Museum of Central Australia, Alice Springs, Northern Territory; **SAM**, South Australia Museum, Adelaide, South Australia, Australia; **USNM**, United States National Museum, Smithsonian Institution, Washington D.C., USA.

**Material examined**

*Genyornis newtoni*: L quadrate SAM P.17124; cranial fragments SAM P.17125; mandible, SAM P10788; sternum SAM P10835; femora: SAM P.13864, SAM P.13878, SAM P.17001- 17012; tibiotarsi: SAM P.13866, SAM P.13927, SAM P.17026- 17035, SAM P.18434; tarsometatarsi: SAM P.13865, SAM P.13879, SAM P.17013-17025; phalanges pes II, III, and IV: e.g. SAM P.17044-17047; scapulocoracoid: SAM P.13872; humerus: SAM P.13871, SAM P.17065, SAM P.17066, SAM P.17068; ulna: SAM P.13873; radius: SAM P.13874, SAM P.17069, SAM P.17070, SAM P.17071; carpometacarpus: SAM P.13875, SAM P.17072; atlas vert: SAM P.13928, SAM P.13929, SAM P.17134B; dorsal vert: SAM P.13935J, SAM P.13935K, SAM P.13935L, SAM P.17092, SAM P.17093, SAM P.17094, SAM P.17095, SAM P.17096, SAM P.17097. Pelvic elements: SAM P.17041 synsacrum, SAM P.17042 synsacrum, SAM P.17048 pt pelvis with R pubis & ischium, SAM P.17049 near complete pelvis. Part skeletons: SAM P.53826, dR tibiotarsus, R tarsometatarsus, R phalanges, dL tibiotarsus, L tarsometatarsus, six articulated & five loose phalanges; SAM P.53827, dL tibiotarsus, L tarsometatarsus, L phalanges (seven artic. & four loose), dR tibiotarsus & R tarsometatarsus articulated with two in situ os sesamoideum intertarsale, a metatarsal ossicle, and phalanges (five articulated + five loose); SAM P.53829, dLR tarsometatarsi & 21 phalanges; SAM P.53830, pt LR tarsometatarsi, articulated pR tibiotarsus & distal R femur, 11L & 11R phalanges, fragment of sternum and rib, pR ulna and radius, R quadrate & part articulated quadratojugal, occipital condyle and three cranial fragments; SAM P.53832, L tarsometatarsus & 10 loose phalanges & metatarsal ossicle, dR tibiotarsus + articulated Os sesamoideum intertarsale, R tarsometatarsus, five articulated & six loose phalanges, one metatarsal ossicle, a fragment of pubis and conjoined ischium; SAM P.53833, Pt pelvis, LR femur, LR tibiotarsi, L tarsometatarsus & phalanges, right half sternum with four articulated sternal ribs, R scapulocoracoid, R humerus, R ulna, R radius, R carpometacarpus, two vertebrae and two thoracic ribs.

*Dromornis planei*: Skull material, see specimens listed in Worthy et al (2016a). Exemplary material listed here. Crania: NTM P9464-106, NTM P9464-109, NTM P9973-6. Mandibles: NTM P2771, symphysis and pt R side; NTM P2772, L side (-cotyla); NTM P2774-3, R side; NTM P9464-112. Premaxillae: NTM P932, NTM P9464-10, NTM P9464-107, NTM P9973-2. Pterygoids: NTM P87103-43, pt L; NTM P9464-101, pt R; NTM P9464-102, pt; NTM P9464-127, L; NTM P9970-1, R. Quadrates: NTM P9464-100, L; NTM P9464-118, L. Axis vertebrae: NTM P907-31, NTM P908. Sternum: NTM P2774. Humeri: NTM P9973-, L. Carpometacarpus: NTM P87103. Pelvis: NTM P87113-?, acetabulum. Femora: NTM P9464-, pL; NTM P9464-193, s+dL; CPC 13844, R; CPC 13845, R. Tibiotarsi: NTM P9464-, dR; NTM P9464-203, dR; NTM P9464-207, pR; NTM P9973-, dR. Tarsometatarsus: NTM P9464-210, dR.

*Dromornis stirtoni*: Skull material, see specimens listed in Worthy et al (2016a); for leg bone material, see material listed in Handley et al. (2016). Other exemplary material as follows. Quadrates: NTM P3201, L; NTM P3202, L; NTM P5401, R. Pterygoids: NTM P3204, R; NTM P3205, pt L; NTM P98114, pt L; NTM P98115, pt L. Mandibles: NTM P3241, left side; NTM P3242, right side; NTM P3245, pt R; NTM P3246, left side; NTM P98107; NTM P98109, left side and symphysis; NTM P98112, left side and symphysis. Premaxillae: NTM P3248, NTM P3253, NTM P3256, NTM P3257, NTM P9245. Humeri: NTM P3206, L; NTM P3208, p+sL; NTM P3209, L; NTM P3210, R; NTM P3211, R; NTM P4784, R; NTM P9623, L. Radius: NTM P3212. Carpometacarpi: NTM P3216, L; NTM P4347, L; SAM P.48645, R; SAM P.48646, pR. Sterni: NTM P3263; NTM P3265; NTM P3266; NTM P3267; NTM P3268; NTM P6005; NTM P9310. Scapulocoracoids: NTM P3275, L; NTM P4471, L. Atlas vertebrae: NTM P3369; NTM P3374. Fused atlas and axis: NTM P3365, NTM P3368. Pelvis: NTM P3434.

*Dromornis murrayi*: see material listed in Worthy et al (2016a).

*Ilbandornis lawsoni*: Cranial fragments, NTM P907-27, NTM P87103-44; quadrates, NTM P3235, P3237; pelvis, NTM P3436; for leg bone material see material listed in Worthy and Yates (2015).

*Ilbandornis woodburnei*: cranial material, NTM P8765-1, P8695-273; QVM: 2000: GFV:20; atlas vertebra, NTM P4497; for leg bone material see material listed in Worthy and Yates (2015).

*Barawertornis tedfordi*: See specimens listed in Worthy et al. (2016a) and Nguyen et al. (2010).

*Gastornis giganteus* (= *Diatryma gigantea*): Eocene Willwood Fm, Wyoming – LACM 6736/18200, distal left tarsometatarsus; LACM 6882/31732, left and right limb bones, vertebrae and ribs; LACM 6879/58874, distal left tarsometatarsus; USNM 15118, part skeleton; AMNH 6169, individual skeleton. Wasatch Fm (early – late Wasatchian 58-52 Ma), Green River Basin, Wyoming – LACM 231/57274, left and right tibiotarsi, tarsometatarsi and vertebrae.

*Gastornis parisiensis*: This taxon was coded from observations of casts of the syntypes of *G. klaasseni* Newton, 1885 (Newton 1886) [now synonymized under *G. parisiensis*] SAM P.48440, right tibiotarsus and SAM P.48441, distal left tibiotarsus; and descriptions (Dollo 1883; Martin 1992; Buffetaut 2008; Angst and Buffetaut 2013; Bourdon, et al. 2016).

*Brontornis burmeisteri*: early – late Miocene (Santacrucian) of Argentina, Province of Santa Cruz. Character coding was by THW from the following specimens: lectotype *B. burmeisteri*, MLP 88-91, left femur, tibiotarsus, MLP 20-91, left tarsometatarsus, Lago Argentina (direct observation); FM-P13259, a complete left and distal right tarsometatarsus, Wreck Flat, 10 miles north of Coy Inlet, from Alvarenga and Höfling (2003) and images by FJD; FM P.15309, distal femur, Rio Gallegos, from images by FJD.

*Patagornis marshi*: This taxon was first scored by THW from the descriptions of NHMUK A-516, a nearly complete skeleton (see detailed descriptions in Ameghino 1895; Andrews, 1899; Alvarenga and Höfling 2003), and AMNH-9264, incomplete skeleton described and illustrated by Sinclair and Farr (1932, Plates XXXII-XXXIV; but see also Alvarenga and Höfling 2003). These were checked and additional characters scored by FJD and FA using observations from the following specimens: AMNH 7005, 7009 and 9264; FM-P13213; MLP 20-85, 20-86, 20-122, 20-143, 20-147, 20-148, 20-151, 20-152, 20-153, 20-154, 20-155, 20-157, 20-158, 20-164, 20-189, 59-XII-14-14 and 84-III-9-21; MPM-PV4242; NHMUK-A516; CORD-Pz1341.

*Wilaru tedfordi*: material as listed in De Pietri et al. (2016).

*Vegavis iaai*: For this analysis, all characters were scored by THW from observations as follows: Holotype (MLP 93-I-3-1) and latex peels taken from it, and from the descriptions by Noriega and Tambussi (1995) and Clarke et al. (2005). These were augmented by observations of the second specimen MACN-PV 19.748 that was recently described by Clarke et al. (2016). Use of both individuals enabled 140 of the 290 characters (~48%) to be scored. However, most cranial characters remain unknown.

*Cariama cristata*: scored by FJD using observations from the following specimens: National Museum of Natural History of the Smithsonian (USNM 11941, 322673, 555731, 019492, 321574, 345199, 430173, 6120030, 630123, 631024, 630286, 631157, 631175 and 631176), Field Museum (FM 105635), Ornithology Collection of the American Museum of Natural History (AMNH 8604), Museo Argentino de Ciencias Naturales Beranrdino Rivadavia (MACN 3717a) and CICTERRA (CIT-O 533).

**1: Master list of Characters (see SI File)**

The character descriptions of Worthy et al. (2016b) were modified as follows.

Five new characters were added:

286. Os sesamoideum intertarsale (within tibial cartilage) presence and form: 0, absent; 1, present and extends across width of the tarsometatarsus-tibiotarsus joint and is much wider than proximodistally long; 2, present and restricted to medial side of the tarsometatarsus-tibiotarsus joint and usually proximodistally elongate. This sesamoid is not known from anseriforms and was found in newly collected articulated material of *Genyornis newtoni* (e.g. SAM P.53827).

287. Cranium, squamosal processus suprameaticus, form: 0, robust , conical, cross-section circular and adpressed to cotyla quadratica oticum caudal to cotyla quadratica squamosum; 1, lateromedially flattened, sliver-like adpressed to cotyla quadratica oticum caudal to cotyla quadratica squamosum; 2, separated laterally from cotyla quadratica oticum and variably flattened. In palaeognaths, the suprameatic area is fused with the processus zygomaticus rather than being a distinct process but here its separation from the cotyla is given priority and so they are coded 2. Character states 0 and 1 typify dromornithids (Worthy et al. 2016a).

288. Syrinx, presence of ossified pessulus (Clarke et al. 2016): 0, absent; 1, present.

289. Syrinx, asymmetry at the tracheobronchial juncture (Clarke et al. 2016): 0, absent; 1, present.

290. Humerus, proximal caudal surface with fossa at the dorsal side of the incisura capitis bounded distally by a transverse crus dorsale fossae and dorsally by the capital shaft ridge: 0, absent; 1, present. Following De Pietri et al. (2016), this structure is treated separately to the fossa pneumotricipitalis dorsalis that aligns parallel to the capital shaft ridge (when it is present), is separated from the fossa pneumotricipitalis ventralis by a proximodistally aligned crus dorsale fossa, and is open distally. Its presence characterises Anhimidae, Presbyornithidae, and *Vegavis*.

In addition, Character 194 was redefined and taxa rescored:

194. Femur, proximal end, caudolateral margin: 0, impressiones obturatoriae on a large bulbous area close to the facies articularis antitrochanterica extending from the lateral onto the caudal facies and the caudolateral margin further distally lacks further prominences; 1, an elevated impressiones obturatoriae close to the facies articularis antitrochanterica and a large prominence further distally on the caudolateral margin for m. ischiofemoralis that is separated from the former by a sulcus as broad as the impression. This character overlaps Bourdon et al. (2009, char. 83). Worthy and Yates (2015) and Worthy et al. (2016b) followed Rich (1979) in assessing the prominence on the caudolateral margin of the shaft in dromornithids as a second insertion area for obturator muscles. However, this interpretation is now seen as unlikely as it does not account for the presence of an impression for the m. ischiofemoralis, which is one of the largest marked impressions laterally on the femur in birds (Hutchinson 2001). Therefore, the large rugose prominence on the caudolateral margin of the shaft and level with the distal end of the trochanter, but slightly proximal to the level of the insertion for m. iliotrochantericus cranialis (which lies distal to the short trochanter in dromornithids), is here interpreted as for the m. ischiofemoralis. In other birds examined, the impression for the m. ischiofemoralis is wholly on the lateral facies and lies separated from the caudolateral margin.

For Character 259, we added a new state (4) to accommodate phorusrhacids:

259. Tarsometatarsus, hypotarsus, number of hypotarsal ridges: 0, four (*Anseranas* and anhimids have two vestigial inner ridges); 1, three ridges (galliforms); 2, two ridges; 3, one centrally located ridge; 4, hypotarsus block-like, no ridges, plantarly flattened, very shallow sulcus slightly towards medial side, steep lateral and medial sides slightly narrowing distally (phorusrhacids).

A few errors were identified and scorings changed as follows: Character 195, *Leipoa* changed to state 1; Character 196, *Megavitiornis* and *Sylviornis* changed to 1; Character 201, *Dinornis* is considered to have only slight shaft curvature so was rescored as 0.

**Higher level relationships of *Vegavis***

*Vegavis iaai* Clarke et al., 2005 is robustly excluded from Anseriformes in all our analyses. It is essentially unresolved as to whether it is the sister group of Anseriformes or the sister group of Gastornithiformes as shown in our Figs 3 and 4. Therefore, *Vegavis* should be recognised as a distinct family and at the ordinal level. *Vegavis* was recently recognised as the type genus of the family Vegaviidae by Agnolin et al. (in press). Vegaviidae is here redefined as volant, leg-propelled diving galloanseres, that have: a mandible with no retroarticular fossae; a vertebral series lacking a notarium; a short, sternally-broad coracoid with a short processus acrocoracoideus that does not project medially, a foramen nervi coracoideum, and a concave cotyla scapularis; scapula with short acromion not projecting cranial to tuberculum coracoideum; an elongate humerus with a shallow, non-pneumatic fossa pneumotricipitalis ventralis, a strongly developed dorsally-directed capital shaft ridge, very elongate crista deltopectoralis, and a distinctive fossa (not homologous to fossa pneumotricipitalis dorsalis) at the dorsal side of the incisura capitis and bound distally by a transverse crus dorsale fossae and dorsally by the capital shaft ridge (shared with Anhimidae and Presbyornithidae); a femur with the insertion scars for m. iliotrochantericus caudalis located at mid depth on lateral facies, a shaft with marked dorsal bowing and lateromedially constricted, and a cranially low crista trochanteris, and an elongate tuber. m. gastrocnemialis lateralis; and tibiotarsus with crista cnemialis cranialis well-developed both cranially and distally, and a cranially flattened shaft. Given Vegaviidae is either the sister taxon to Gastornithiformes or Anseriformes, it is recognised here as representative of the new order Vegaviiformes.

**Phylogenetic analyses: ADDITIONAL DESCRIPTION**

**BAYESIAN ANALYSES**

The morphological, stratigraphic, size and diet data were simultaneously analysed using tip-dated Bayesian analyses (Drummond and Suchard 2010; Ronquist et al. 2012; Gavryushkina et al. 2017). The optimal evolutionary histories that explain all three data sources were inferred using Markov-chain Monte Carlo (MCMC) approaches as implemented in the BEAST 1.8.4 package with summary statistics and trees generated via LogCombiner and TreeAnnotator (Drummond et al. 2012). Inferred ancestral states for size and diet were recorded for each node (with diet inferred for each fossil tip), for each sampled tree. BEAST1 was used because it is increasingly widely employed for tip-dated phylogenetic analysis of time-sampled data including fossils (e.g. Drummond and Suchard 2010; Gavryushkina et al. 2017) and can incorporate and reconstruct ancestral states for continuous as well as discrete traits (e.g. Lee et al. 2014). The executable BEAST xml files, with annotations describing model, prior, MCMC and logging settings, are in SI. The description below summarises the major points.

In all Bayesian analyses, the morphological characters were analysed using the Mk-model correcting for non-sampling of constant characters (Lewis 2001; Alekseyenko et al. 2008), which has been extensively evaluated with generally favourable results (Wright and Hillis 2014; O'Reilly et al. 2016). Polymorphic morphological data (0&1) could be treated exactly as coded (e.g. 0 or 1 but *not* 2), not as total uncertainty (0 or 1 or 2): an improvement over earlier versions of BEAST. 60 anatomical characters forming morphoclines were treated as ordered (see list in Worthy et al. 2016b); diet was also ordered. To ensure all state transitions were weighted identically, even for morphological characters with different numbers of observed states, a 5-state substitution Lewis model was employed for all traits. Partitioning characters according to the number of observed states can result in better model fit but has minimal effect on phylogeny, dates or ancestral state reconstruction (e.g. Gavryushkina et al. 2017; King et al. 2017). Furthermore, such state-partitioned models have potential drawbacks: (1) They increase the influence of characters with more states (e.g. King et al 2017); (2) Unless homoplasy is exceptionally rampant, the observed states of a character will not represent all possible states (Hoyall Cuthill 2015). Indeed, in related non-penguin outgroups (Ksepka et al. 2012), increased state-space can be observed for many of the penguin characters modelled in Gavryushkina et al. (2017). As a further example, most sites in amino acid alignments will not exhibit all 20 possible states, and many DNA alignments will have sites with fewer than four states. Yet, amino acid or DNA alignments are not typically analysed by assuming that the observed states at each variable position are the *only* possible states.

The continuous trait (size) was analysed using constant-rate and variable-rate Brownian motion models (see below). Analyses using directional models did not result in better fit, probably due to the comparatively sparse fossil and taxon sampling.

Bayes Factors (*sensu* Kass and Raftery 1995) calculated using stepping-stone sampling (Xie et al. 2011) found significant variation in rates of morphological evolution across different characters, favouring the inclusion of the gamma parameter (Yang 1994) for among-character rate variation (Bayes Factor ~210). There was also significant variation in rates of evolution across different lineages, with relaxed (uncorrelated lognormal: Drummond et al. 2006) clocks preferred over a strict clock (BF ~123). Thus, all Bayesian analyses employed the gamma parameter and relaxed clocks.

The most appropriate available tree prior (birth-death serial-sampling: Stadler 2010) in BEAST 1.8 was used. Tip calibrations were employed using the full stratigraphic range for each taxon, with each taxon's age being represented by a uniform prior spanning min-max ages. A soft constraint on root age (crown Aves) was employed, with the 95% interval based on the range found in Prum et al. (2015: their analysis with *Vegavis* included): normal prior with mean of 78.95 and sd of 3.14.

The Bayesian analyses were performed with, and without, a molecular backbone; the latter approach has not previously been implemented in BEAST 1 and the relevant code is appended in the xml file. Without any root constraint, the non-backbone analyses had trouble converging on a sensible rooting. Thus, we implemented a root constraint which reflected the most sensible root consistent with the corresponding parsimony analyses (between ratites and neognaths); to avoid prejudicing the position of other taxa such as lithornithids and tinamous, these taxa were not included in the root constraint and were free to move as the data dictated.

**Character support for our preferred BEAST analysis**

Morphological characters were optimised (using parsimony as implemented in PAUP: Swofford 2003) on the Bayesian MCC consensus tree, to identify synapomorphies for retrieved nodes.

**Neognathae**. No unambiguous apomorphies characterise the neognath stem. However, apomorphic changes in five characters, narrowly separated (not widely separated) tuba auditiva communis (Char. 22, 0-->1), mediolaterally narrow (not wide) vomer (Char. 39, 0-->1), os palatinum and os pterygoideum not fused (unfused, Char. 41, 0-->1), pelvis with closed (not open) foramen ilioischiadicum caudally (Char. 183, 0-->1), and syrinx with an ossified pessulus (Char. 288, 0-->1) potentially characterise stem Neognaths (Acctran & Deltran). Char. 15, cranium with open (versus closed) frontoparietal suture no longer optimises as primitive state for palaeognaths, as it did under parsimony when ratites were removed.

**Galloanseres**. Seven unambiguous apomorphies characterise the stem of Galloanseres in both Acctran and Deltran, of which Char. 20 (0==>1, CI=0.500, recessus tympanicus becoming bound ventrally by laterally projecting ala parasphenoidalis which links caudally to the proc. paroccipitalis), Char. 28 (0==>1, CI= 0.667, position of proc. basipterygoidei anterior to basitemporal platform), Char. 60 (0==>1, CI= 1.000, mandible with three cotylae fossae articularis) are most compelling.

**Gastornithiformes**. 13 unambiguous apomorphies characterise the clade Gastornithiformes in both Acctran and Deltran, of which Char. 63 (0==>2, CI=0.667, depth regio coronoidei of mandible much less than depth at angulus mandibulae at posterior end dentary) is the most compelling.

***Vegavis***. *Vegavis* is typified by 25 unambiguous apomorphies in both Acctran and Deltran, but none are autapomorphic.

**Anseriformes**. Anseriforms (exclusive of *Vegavis*) are supported by five unambiguous apomorphies, many fewer than under Parsimony with ratites removed. The clade of presbyornithids and Anatoidea (Anseranatidae + Anatidae) is strongly supported by 10 unambiguous apomorphies in both Acctran and Deltran, of which Char. 28 (1==>2, CI=0.667, proc. basipterygoidei on sides of rostrum parasphenoidale anterior to caudal end of rostrum), Char. 65 (0==>1, CI=1.000, mandible with recessus conicalis), and Char. 69 (0==>1, CI=1.000, mandible rami laterally compressed defining long narrow inter-ramal area), are most compelling. Anatoidea exclusive of presbyornithids are supported by 11 unambiguous apomorphies in both Acctran and Deltran, of which Char. 63 (0==>1, CI=0.667, mandible, depth regio coronoidei is markedly deeper (>1.5x depth) than that of the posterior dentary) is the most compelling.

**Galliformes.** Galliformes, inclusive of Sylviornithidae, are supported by 11 unambiguous apomorphies, and exclusive of Sylviornithidae by 11 unambiguous apomorphies, of which Char. 3 (2==>0, CI=0.500, premaxilla with divergent rami) is the most compelling.

**Neoaves**. Neoaves are supported by seven unambiguous apomorphies, of which Char. 35 (0==>1, CI=1.000, crista ventralis of palatines strongly developed ventrally), and Char. 159 (1==>0, CI=0.500, carpometacarpus, proximal end, caudal view, minor metacarpal extends dorsad of ventral rim of trochlea carpalis in synostosis with os metacarpale majus) are the most compelling.

**Palaeognaths exclusive of Lithornithids**. Crown group palaeognaths are supported by 14 unambiguous apomorphies in both Acctran and Deltran, of which none are compelling.

See ‘Worthy et al Gallo48_290_BEAST_apolist_log.txt’ in SI for the complete apomorphy list.

**Credits for Use of images from Phylopic.org for the silhouettes in Figs. 1 and 2.**

*Vegavis* and *Gastornis* by Matt Martynuik, licence <https://creativecommons.org/licenses/by/3.0/>;
*Ortalis* by anonymous artist, licence Public Domain Mark 1.0;
Dromornithid by Zimices, licence <https://creativecommons.org/licenses/by/3.0/>;
*Lithornis* and *Cariama* by George Edward Lodge modified by T. Michael Keesey, licence Public Domain Mark 1.0;
*Anser* by Ferran Sayol; Public Domain Dedication 1.0;

*Tinamus major* by George Edward Lodge, licence Public Domain Mark 1.0;
*Struthio* by Darren Naish (vectorise by T. Michael Keesey) licence, <https://creativecommons.org/licenses/by/3.0/>;
*Gallus gallus* and *Paraphysornis* (for *Patagornis*) by Steven Traver licence. Public Domain Dedication 1.0;

The palaeomap in the Graphical Abstract was downloaded from <http://www.odsn.de/odsn/services/paleomap/paleomap.html>

**Additional Files**

**1. The complete annotated list of characters for the analysis.**

SI Master Characters for galloanseres May 2017.docx

**2. The executable file for Parsimony analyses using PAUP (for Figure 3)**

Worthy et al Galloansere nexus ratites excluded.nex

**3. The executable file for the Beast analysis (for Figure 4):**

Worthy et al BEAST file Gallo48_290asc_Ord_uclnDis_uclnCont.xml

**4-6. Apomorphy lists from the parsimony and BEAST analyses for Figures 2, 3 and 4.**

Worthy et al Gallo 48taxa_290_PAUP_apolist_log.txt

Worthy et al Gallo45_ratites_removed_290_PAUP_apolist_log.txt

Worthy et al Gallo48_290_BEAST_apolist_log.txt

**7. Excel file with mass, diet and stratigraphic age:**

Worthy et al data on mass diet and age.xlsx

**References in this Supplementary Information**

Agnolin, F.L., Brissón Egli, F., Novas, F., García Marsá, J., and Chatterjee, S. In press. Vegaviidae, a new clade of Southern diving birds that survived the K/T boundary. Science of Nature Naturwissenchaften,

Alekseyenko, A.V., Lee, C., Suchard, M.A., 2008. Wagner and Dollo: a stochastic duet by composing two parsimonious solos. Systematic Biology 57, 772–784.

Alvarenga, H., Höfling, E., 2003. Systematic revision of the Phorusrhacidae. Papéis Avulsos de Zoologia 43, 55–91.

Ameghino, F., 1895. Sur les oiseaux fossiles de Patagonie. Boletín del Instituto Geográfico de Argentina 15, 501–602.

Andrews, C., 1899. On the extinct birds of Patagonia. I, The skull and skeleton of *Phororhacos inflatus* Ameghino. Transactions of the Zoological Society of London 15, 55–86.

Angst, D., Buffetaut, E., 2013. The first mandible of *Gastornis* Hébert, 1855 (Aves, Gastornithidae) from the Thanetian (Paleocene) of Mont-de-Berru (France). Revue de Paléobiologie 32, 423–432.

Bourdon, E., de Ricqlès, A., Cubo, J., 2009. A new transantarctic relationship: morphological evidence for a Rheidae-Dromaiidae-Casuariidae clade (Aves: Palaeognathae, Ratitae). Zoological Journal of the Linnean Society 156, 641–663, doi:10.1111/j.1096-3642.2008.00509.x.

Bourdon, E., Mourer-Chauviré, C., Laurent, Y., 2016. Early Eocene birds from La Borie, southern France. Acta Palaeontologica Polonica 61, 175–190.

Buffetaut, E., 2008. First evidence of the giant bird *Gastornis* from southern Europe: a tibiotarsus from the Lower Eocene of Saint-Papoul (Aude, southern France). Oryctos 7, 75–82.

Clarke, J.A., Chatterjee, S., Li, Z., Riede, T., Agnolin, F., Goller, F., Isasi, M.P., Martinioni, D.R., Mussel F.J., Novas, F.E., 2016. Fossil evidence of the avian vocal organ from the Mesozoic. Nature 538(7626), 502–505, doi:10.1038/nature19852.

Clarke, J.A., Tambussi, C.P, Noriega, J.I., Erickson, G.M., Ketcham, R.A., 2005. Definitive fossil evidence for the extant avian radiation in the Cretaceous. Nature 433(7023), 305–308.

De Pietri, V.L., Scofield, R.P., Zelenkov, N., Boles, W.E., Worthy, T.H., 2016. The unexpected survival of an ancient lineage of anseriform birds into the Neogene of Australia: the youngest record of Presbyornithidae. Royal Society Open Science 3, 150635 [16 pp], doi:10.1098/rsos.150635.

Dollo, L., 1883. Note sur la présence du *Gastornis edwardsii* Lemoine dans l’assise inférieure de l’étage landénien à Mesvin, près Mons. Bulletin du Musée Royal d’Histoire Naturelle de Belgique 2, 297–305.

Drummond, A.J., Ho, S.Y.W., Phillips, M.J., Rambaut, A., 2006. Relaxed phylogenetics and dating with confidence. PLoS Biol. 4, e88. (doi:10.1371/journal.pbio.0040088)

Drummond, A.J., Suchard, M.A., 2010. Bayesian random local clocks, or one rate to rule them all. BMC Biology 8, 114, doi:10.1186/1741-7007-8-114.

Drummond, A.J., Suchard, M.A., Xie, D., and Rambaut, A., 2012. Bayesian phylogenetics with BEAUti and the BEAST 1.7. Molecular Biology and Evolution 29, 1969–1973.

Gavryushkina, A., Heath, T.A., Ksepka, D.T., Stadler, T., Welch, D., Drummond, A.J., 2017. Bayesian total-evidence dating reveals the recent crown radiation of penguins. Systematic Biology 66, 57–73

Handley, W.D., Chinsamy, A., Yates, A.M., Worthy, T.H., 2016. Sexual dimorphism in the late Miocene mihirung *Dromornis stirtoni* (Aves: Dromornithidae) from the Alcoota Local Fauna of central Australia. Journal of Vertebrate Paleontology 36(5), e1180298 [21 pp], doi:10.1080/02724634.2016.1180298.

Hoyall Cuthill, J.F., 2015. The morphological state space revisited: what do phylogenetic patterns in homoplasy tell us about the number of possible character states? Interface Focus 5(6): 20150049; doi:10.1098/rsfs.2015.0049.

Hutchinson, J.R., 2001. The evolution of femoral osteology and soft tissues on the line to extant birds (Neornithes). Zoological Journal of the Linnean Society 131, 169–197.

Kass, R.E., Raftery, A.E., 1995. Bayes factors. Journal of the American Statistical Association 90, 773–795.

King, B., Qiao, T., Lee, M.S.Y., Min, Z., Long, J.A., 2017. Bayesian morphological clock methods resurrect placoderm monophyly and reveal rapid early evolution in jawed vertebrates. Systematic Biology, 66(4), 499-516, doi:10.1093/sysbio/syw107.

Ksepka, D.T., Fordyce, R.E., Ando, T., Jones, C.M., 2012. New fossil penguins (Aves, Sphenisciformes) from the Oligocene of New Zealand reveal the skeletal plan of stem penguins. Journal of Vertebrate Paleontology 32, 235–254.

Lee, M.S.Y., Cau, A., Naish, D., Dyke G. J. 2014. Sustained miniaturisation and evolutionary novelty in the dinosaurian ancestors of birds. Science 345, 562–566.

Lewis, P.O., 2001. A likelihood approach to estimating phylogeny from discrete morphological character data. Systematic Biology 50, 913–25.

Martin, L.D., 1992. The status of the late Paleocene birds *Gastornis* and *Remiornis*. In: Campbell, K.E. (Ed.), Papers in Avian Paleontology honoring Pierce Brodkorb. Natural History Museum of Los Angeles County Science Series 36, 97–108.

Newton, E.T., 1885. *Gastornis klaasseni* Newton, a gigantic bird from the Lower Eocene of Croydon. Geological Magazine 3, 362–364.

Newton, E.T., 1886. On the remains of a gigantic bird (*Gastornis klaasseni*, n. sp.) from the Lower Eocene beds near Croydon. Transactions of the Zoological Society of London 12, 143–160.

Noriega, J.I., Tambussi, C.P., 1995. A Late Cretaceous Presbyornithidae (Aves: Anseriformes) from Vega Island, Antarctic Peninsula: Paleobiogeographic implications. Ameghiniana 32, 57–61.

Prum, R.O., Berv, J.S., Dornburg, A., Field, D.J., Townsend, J.P., Lemmon, E.M., Lemmon, A.R., 2015. A comprehensive phylogeny of birds (Aves) using targeted next generation DNA sequencing. Nature 526, 569–73.

O'Reilly, J.E., Puttick, M.N., Parry, L., Tanner, A.R., Tarver, J.E., Fleming, J., Pisani, D., Donoghue, P.C.J., 2016. Bayesian methods outperform parsimony but at the expense of precision in the estimation of phylogeny from discrete morphological data. Biology Letters 12(4), 20160081, doi:10.1098/rsbl.2016.0081.

Rich, P.V., 1979. The Dromornithidae, an extinct family of large ground birds endemic to Australia. Bureau of National Resources, Geology and Geophysics Bulletin 184, vii + 1–194.

Ronquist, F., Klopfstein, S., Vilhelmsen, L., Schulmeister, S., Murray, L., Rasnitsyn, A.P., 2012. A total-evidence approach to dating with fossils, applied to the early radiation of the Hymenoptera. *Systematic Biology* 61, 973–999, doi:10.1093/sysbio/sys058.

Sinclair, W., Farr, M., 1932. Aves of the Santa Cruz beds. In: Scott, W. (Ed.) Reports of the Princeton University expeditions to Patagonia (1896-1899). Princeton University. 7, 157–191.

Stadler, T., 2010. Sampling-through-time in birth-death trees. Journal of Theoretical Biology 267, 396–404, doi:10.1016/j.jtbi.2010.09.010.

Swofford, D.L., 2003. *PAUP*.* Phylogenetic Analysis Using Parsimony (*and Other Methods). Version 4. Sinauer Associates, Sunderland, MA.

Worthy, T.H., Yates, A., 2015. Connecting the thigh and foot: resolving the association of post-cranial elements in the species of *Ilbandornis* (Aves: Dromornithidae). Alcheringa 39(3), 407–427, doi:10.1080/03115518.2015.1015818.

Worthy, T.H., Handley, W.D., Archer, M., Hand, S.J., 2016a. The extinct flightless mihirungs (Aves: Dromornithidae): cranial anatomy, a new species and assessment of Oligo-Miocene lineage diversity. Journal of Vertebrate Paleontology 36(3), e1031345 (21 pages), doi:10.1080/02724634.2015.1031345.

Worthy, T.H., Mitri, M., Handley, W.D., Lee, M.S.Y., Anderson, A., Sand, C., 2016b. Osteology supports a stem-Galliform affinity for the giant extinct flightless bird *Sylviornis neocaledoniae* (Sylviornithidae, Galloanseres). PLoS ONE 11(3), e0150871 (62 pp); doi:10.1371/journal.pone.0150871.

Wright, A.M., Hillis, D.M., 2014. Bayesian analysis using a simple likelihood model outperforms parsimony for estimation of phylogeny from discrete morphological data. PLoS ONE 9, e109210.

Xie, W., Lewis, P.O., Fan, Y., Kuo, L., Chen, M.-H., 2011. Improving marginal likelihood estimation for Bayesian phylogenetic model selection. Systematic Biology 60, 150–160.

Yang, Z.H., 1994. Maximum likelihood phylogenetic estimation from DNA sequences with variable rates over sites: approximate methods. Journal of Molecular Evolution 39, 306–314.
